# Multiscale investigation of mealiness in apple: an atypical role for a pectin methylesterase during fruit maturation

**DOI:** 10.1186/s12870-014-0375-3

**Published:** 2014-12-31

**Authors:** Sandrine Mikol Segonne, Maryline Bruneau, Jean-Marc Celton, Sophie Le Gall, Mathilde Francin-Allami, Marjorie Juchaux, François Laurens, Mathilde Orsel, Jean-Pierre Renou

**Affiliations:** AgroCampus-Ouest, UMR 1345 Institut de Recherche en Horticulture et Semences, F-49045 Angers, France; INRA, UMR 1345 Institut de Recherche en Horticulture et Semences, F-49071 Beaucouzé, France; Université d’Angers, UMR 1345 Institut de Recherche en Horticulture et Semences, SFR 4207 QUASAV, PRES L’UNAM, F-49045 Angers, France; INRA, UR1268 Biopolymères, Interactions, Assemblages, F-44316 Nantes, France

**Keywords:** Apple, Cell wall, *Malus domestica*, PME, Fruit texture, Transcriptome

## Abstract

**Background:**

Apple fruit mealiness is one of the most important textural problems that results from an undesirable ripening process during storage. This phenotype is characterized by textural deterioration described as soft, grainy and dry fruit. Despite several studies, little is known about mealiness development and the associated molecular events. In this study, we integrated phenotypic, microscopic, transcriptomic and biochemical analyses to gain insights into the molecular basis of mealiness development.

**Results:**

Instrumental texture characterization allowed the refinement of the definition of apple mealiness. In parallel, a new and simple quantitative test to assess this phenotype was developed.

Six individuals with contrasting mealiness were selected among a progeny and used to perform a global transcriptome analysis during fruit development and cold storage. Potential candidate genes associated with the initiation of mealiness were identified. Amongst these, the expression profile of an early down-regulated transcript similar to an *Arabidopsis thaliana* pectin methylesterase gene (*AtPME2*) matched with mealiness development. *In silico* analyses of this *Malus x domestica PME* gene (*MdPME2)* confirmed its specific pattern compared with all other identified *MdPME* genes. Protein fusion experiments showed that MdPME2 is secreted into the apoplast in accordance with a possible activity on pectin structure. Further microscopic analysis indicated a progressive loss of cell to cell adhesion in mealy apple fruits. Biochemical analysis revealed specific modifications of pectin residues associated with mealiness, without global changes in the degree of methylesterification of pectins.

**Conclusions:**

These data support the role of PME in cell wall remodelling during apple fruit development and ripening and suggest a local action of these enzymes. Mealiness may partially result from qualitative and spatial variations of pectin microarchitecture rather than quantitative pectin differences, and these changes may occur early in fruit development. The specific *MdPME2* gene highlighted in this study could be a good early marker of texture unfavourable trait in apple.

**Electronic supplementary material:**

The online version of this article (doi:10.1186/s12870-014-0375-3) contains supplementary material, which is available to authorized users.

## Background

Apple is amongst the largest consumed fruit in the world. During fruit maturation and ripening, apples undergo important metabolic changes consisting in the conversion of starch to simple sugars, the reduction in organic acid contents, skin colour changes, production of flavoured volatiles and flesh softening [1]. In some genotypes, mealiness may occur during storage. This modification in texture, often associated with excessive softening, makes apple fruits less attractive to consumers [2] and increases costs for marketers due to a loss of quality and a higher susceptibility to pathogens [3,4].

Mealiness results from an undesirable ripening process and is characterized by textural deterioration, resulting in soft, grainy and dry fruit [5,6]. Many reports about apple texture modifications during ripening have focused either on soft or mealy phenotypes. Iwanami *et al.* [7] underlined the coexistence of both phenotypes, suggesting that softening studies should consider the degree of mealiness to adequately investigate apple ripening process. Microscopic observations led to the conclusion that mealiness is associated with the dissolution of the middle lamella and the disruption of the primary cell wall [5]. The authors suggested that these changes of the cell wall results in a loss of cell-to-cell adhesion, resulting in tissue fracture by cell-to-cell separation rather than by cell rupture during mastication of the fruit. Fruit cell walls are a highly complex matrix composed of microfibrils of cellulose embedded in a network of hemicelluloses, pectins and proteins [8]. Amongst the genetically-programmed biochemical events which occur during fruit ripening, cell wall modifications are the most described. Many enzymes are involved in the dynamics of the cell wall during fruit development and ripening [9,10]. High α-L-arabinofuranosidase gene expression and enzyme activity levels have been reported as being related to mealiness [11]. Pectin methylesterase (PME) activity has been shown to be higher during the early stage of ripening in soft apple fruits [12]. Both enzymes act on pectins (rhamnogalacturonan-I and homogalacturonan respectively) which render the cell wall matrix more accessible to other degrading enzymes such as polygalacturonases (PGs) [12,13]. Experiments with transgenic apple plants showed that PG1 activity is necessary for “Royal Gala” softening [14]. Despite several studies, little is known about mealiness development and the associated molecular events. Nevertheless, all reports have concluded that fruit ripening results from a complex mechanism that cannot be reduced to a single gene but rather involves a complex network. Moreover it is likely that mealiness as a unique sensory parameter reports various biochemical and structural situations in different fruit species.

Transcriptome analyses have the potential to screen many gene pathways simultaneously. In recent years, a number of studies have integrated these extensive data sets in order to investigate apple fruit ripening. Most of them were based on ‘custom made’ microarrays developed from EST apple databases. Lee *et al.* [15] used a 3,484 cDNAs array to identify 192 cDNAs involved in the early stages of apple fruit development. Janssen *et al.* [16] undertook a similar study on “Royal Gala” and extended to the fruit ripening stages, using a 13,000 gene apple oligonucleotide array. This same array was used previously to identify 17 putative candidate genes regulated by ethylene and involved in the production of aroma compounds in the fruits of transgenic lines of “Royal Gala” [17]. Costa *et al.* [18] focused on the ripening process of “Mondial Gala” using another apple EST array and heterologous hybridization using a tomato array. A high density 23 k long-oligo apple array was designed by Zhu *et al.* [19] to compare transcriptome profiles between two cultivars with contrasted firmness and crispness phenotypes at harvest. Distinct sets of genes from various metabolic pathways were classified as cultivar dependent and may contribute to the observed phenotypic variation such as xyloglucan endo-transglycosylase/hydrolase (XTH) genes involved in cell wall remodelling [10]. Other apple microarrays were reported on apple tree scions [20] and fruitlet abscission [21]. Until now, all published apple transcriptome studies were restricted to a subset of identified transcripts. Due to the availability of the apple genome sequence [22], the AryANE_v1 microarray representing the fully annotated genome was recently designed [23].

The results presented in this study constitute the first whole genome analysis of apple and provide insight into molecular regulatory events involved in fruit mealiness development, which is considered as the development of an unfavourable texture trait. Up to now, the most commonly used method to characterize mealiness was based on sensory analyses. As this method is rather time consuming and can be user dependent, a novel simple cell cohesion test was developed to assess mealiness. In addition, using the AryANE_v1 microarray, six siblings from a population of hybrids with contrasting phenotypes for fruit mealiness were studied. As modifications leading to mealiness are probably initiated early in ripening, the expression profiles were studied over the complete fruit cycle from cell expansion to fruit cold storage. Amongst all the differentially expressed genes, only a few displayed consistent and opposite expression profiles between all mealy and non-mealy genotypes leading to the identification of one *Malus x domestica* member of the pectin-methylesterase (EC 3.1.1.11) gene family. Investigation through biochemical analyses suggests an essential role in mealiness development.

## Results

### Sensory evaluation of fruit mealiness during storage

Apple texture including mealiness was evaluated by a panel of experts from harvest to four months of cold storage. Mealiness assessment is based on the sensation of fruit dry flesh associated with a grainy texture. It is a genetically determined trait also dependant on environmental conditions [6]. In order to identify stable fruits phenotypes over the years, notations were repeated for four consecutive years and led to the selection of six hybrids (Table 1). These hybrids are full-sibs from a population segregating for mealiness [11,24]. The three hybrids M16, M20 and M49 produced non-mealy apples while the three hybrids M40, M48 and M74 displayed mealy apples after cold storage. Their degree of mealiness increased greatly between two and four months of cold storage. Nonetheless, a slight influence of the production year was observed on mealiness notations. In 2011, apple fruits became mealy earlier displaying higher score at 60 and 120 days after harvest (DAH) when compared with the other years. On the opposite, in 2010 the mealy hybrid M40 displayed a lowest level of mealiness at 120 DAH than the other years (Table 1). It is noteworthy that M48 was always less mealy than the 2 others hybrids M40 and M74.

**Table 1:** **Sensory evaluation of mealiness for individual hybrids**

	**2007**			**2009**			**2010**			**2011**			**Mean**
	**H**	**60DAH**	**120DAH**	**H**	**60DAH**	**120DAH**	**H**	**60DAH**	**120DAH**	**H**	**60DAH**	**120DAH**	**120 DAH**
**M16**	1.0	1.0	1.5	1.0	1.0	1.0	1.0	1.0	1.0	1.0	1.0	1.3	1.2
**M20**	1.0	1.0	1.0	1.0	1.0	1.0	1.0	1.5	1.5	1.0	1.0	1.3	1.2
**M49**	1.0	1.0	1.3	1.0	1.0	1.0	1.0	1.3	1.1	1.0	1.0	1.5	1.2
**M40**	2.0	1.5	4.3	1.5	2.8	4.0	1.2	2.0	2.4	1.0	3.5	4.5	3.8
**M48**	1.0	2.0	2.8	1.0	2.5	2.5	1.0	1.3	2.2	1.0	2.3	3.0	2.6
**M74**	1.0	1.0	3.5	1.5	2.0	3.0	1.3	3.0	3.4	1.0	4.0	4.5	3.6

Scores range from 1 (no mealiness perception) to 5 (very high mealiness perception). Evaluations were carried out during four years (2007 and from 2009 to 2011) at three time points (H: harvest, 60 and 120 DAH: i.e. 2 and 4 months of storage in a cold chamber at 1°C). “Mean 120 DAH” is the mean of the four years for the last time point. Mealy hybrids are highlighted in grey.

### Fruit texture instrumental phenotype

**Figure 1:**
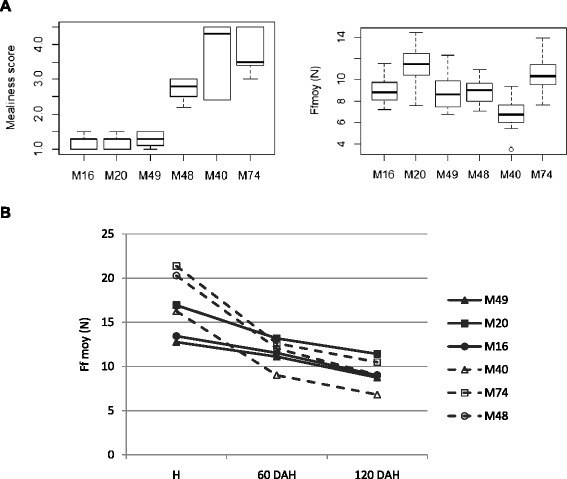
**Fruit firmness for individual hybrids evaluated by penetrometry. A** Comparison between sensory and instrumental measurements at 120 DAH. The hybrids are ordered in accordance with their sensory profiles (M16, M20, M49 as non-mealy and M48, M40 and M74 as mealy). Ffmoy, expressed in Newton, represents the mean force measured in the apple flesh (mean of forces measured at 7 and 9 mm of deformation). Boxplots present the median (the central mark), the region between the upper and lower quartiles (white area), the 95% confidence intervals around the median (the whiskers), the outliers are plotted individually. **B** Lost of flesh firmness from harvest to 120 DAH. The dash lines are associated with mealy hybrids and the bold ones with non-mealy.

Measurement of fruit firmness on unpeeled apples using a penetrometer revealed no difference between mealy and non-mealy apple fruits at 120 DAH (Figure 1A). Mealiness is therefore not correlated with the measurement of firmness in this study. However, the rate of softening, measured as the loss of firmness, between H and 60DAH, was different between mealy and non-mealy apple fruits (Figure 1B).The measured force decreased by 42.1% between harvest and 60 DAH for the mealy apples, and in contrast by only 16.5% for the non-mealy apples.

In order to assess fruit mealiness with an alternative method to sensory analysis, a cell cohesion test was developed. This test is based on the hypothesis that fruit mealiness results from cell detachment rather than cell wall disruption during mastication. From the mealiness evaluation of 158 apples fruits (extended to other genotypes including “Golden Delicious”), a significant positive correlation was observed between sensory analysis and detached cell quantification (Pearson coefficient correlation = 0.83, P-value < 0.01) (Additional file 1). This result confirmed the hypothesis that parenchymal cells of mealy apple fruits are less strongly bound to each other than cells from non-mealy apple fruits.

**Figure 2:**
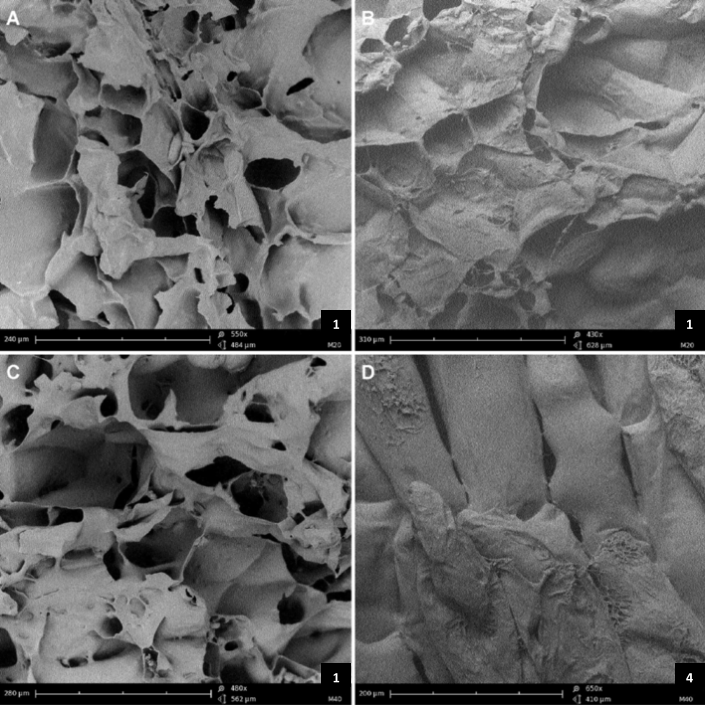
**Cryo-scanning electron micrographs of fractured apple parenchymes in 2012. (A-C)** at harvest; **(B, D)** at 120 DAH, after 4 months of cold storage at 1°C. **(A, B)** M20, a non-mealy hybrid; **(C, D)** M40, a mealy hybrid. Bar scale is at the bottom-left corner of each micrograph, score of mealiness is the number at the bottom-right corner.

This hypothesis was further confirmed using SEM imaging of parenchyma surfaces obtained from fractured apple flesh. Representative images of mealy and non-mealy apple fruits phenotyped at harvest and 120 DAH are shown Figure 2. At harvest (Figure 2A-C), the fracture pattern of apples from all hybrids is characterized by the disruption of individual cells and the presence of air gaps. At 120 DAH, the fracture pattern of non-mealy samples (Figure 2B) presented a similar pattern to that observed at harvest, with burst cells. In contrast, mealy fruit patterns at 120 DAH were characterized by a palissadic arrangement of the cells and minimal cell rupture (Figure 2D). In accordance with the hypothesis, mealy apple tissues were fractured by cell detachment rather than by cell rupture.

### Transcriptome characteristics of mealy apples

**Figure 3:**
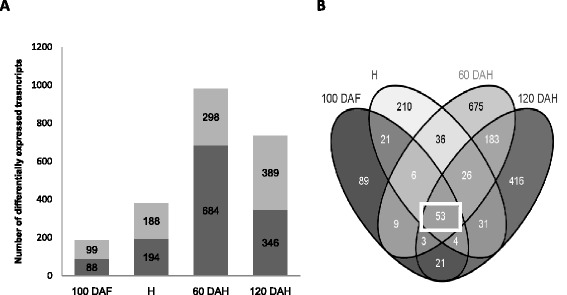
**Distribution of differentially expressed genes between mealy and non-mealy hybrids during kinetic of fruit maturation. A** Histogram illustrating the number of significant differentially expressed genes between mealy (M74, M40) and non-mealy (M20, 49) hybrids during the fruit kinetic. In dark gray, genes are up-regulated in non-mealy hybrids; in soft grey, they are up-regulated in mealy hybrids. The number of genes per groups is shown. **B** Venn diagrams showing the overlap among kinetic time-point of all deregulated genes between mealy and non-mealy hybrids. The square points out the 53 genes deregulated during all kinetic.

In order to characterize the transcriptome of mealy apples, the four genotypes which displayed the strongest phenotypic differences were studied as mealy (M40, M74) *versus* non-mealy (M49, M20) pairs over the developmental (100 DAF, H) and storage (60 DAH, 120 DAH) periods during several years. The expression profiles of M40 and M74 apples were respectively compared to the expression profiles of M49 and M20 apples. Differentially expressed genes were identified with significant P-values for paired sample t-tests (P-value < 0.01). We hypothesized that genes involved in mealiness displayed a differential expression pattern for each pair of mealy/non-mealy hybrids. Genes were selected only when they displayed identical expression patterns for both pairs (up or down regulated for both pairs) for at least one time point. Thus, a total of 1783 differentially expressed transcripts were selected at one or more developmental stages. The number of differentially expressed transcripts increased in concert with mealiness development: the main phenotypic changes were initiated about 60 DAH which was also the time point displaying the highest transcription deregulation (Figure 3A). Based on MDP genes annotations, the differentially expressed transcripts were classified into functional categories (Additional file 2). The most represented functions were linked to protein modifications (11%, Wilcoxon test: P < 0.05), transcriptional regulation (5.6%, P = 0.09), biotic stress responses (4.6%, P < 0.05), photosynthesis (3.2%, P < 0.05), cell wall modification (1.9%,P < 0.05), and unknown functions (33.8%, P < 0.05). Moreover, sense (S) and anti-sense (AS) probes were designed for each annotated apple CDS, and 13.6% of the differentially expressed probes corresponded to AS transcripts. Celton *et al*. [23] demonstrated relatively high levels of AS transcription in apple fruits and seeds compared with other organs. This should be considered for further studies on fruit development as these AS transcripts are likely to be involved in small interfering RNA (siRNA) dependent negative regulation of the coding mRNAs.

In order to validate the microarray data, the relative transcript abundances of a subset of differentially expressed genes were tested by RT-qPCR, using cDNA from mealy and non-mealy apples. The results were in agreement with those obtained from the microarrays analysis (Additional file 3). The observations of gene expression level differences between mealy and non-mealy apple fruits were similar with both techniques (Pearson correlation coefficient = 0.86, P-value < 0.01).

### Constitutively deregulated genes associated with mealiness

**Figure 4:**
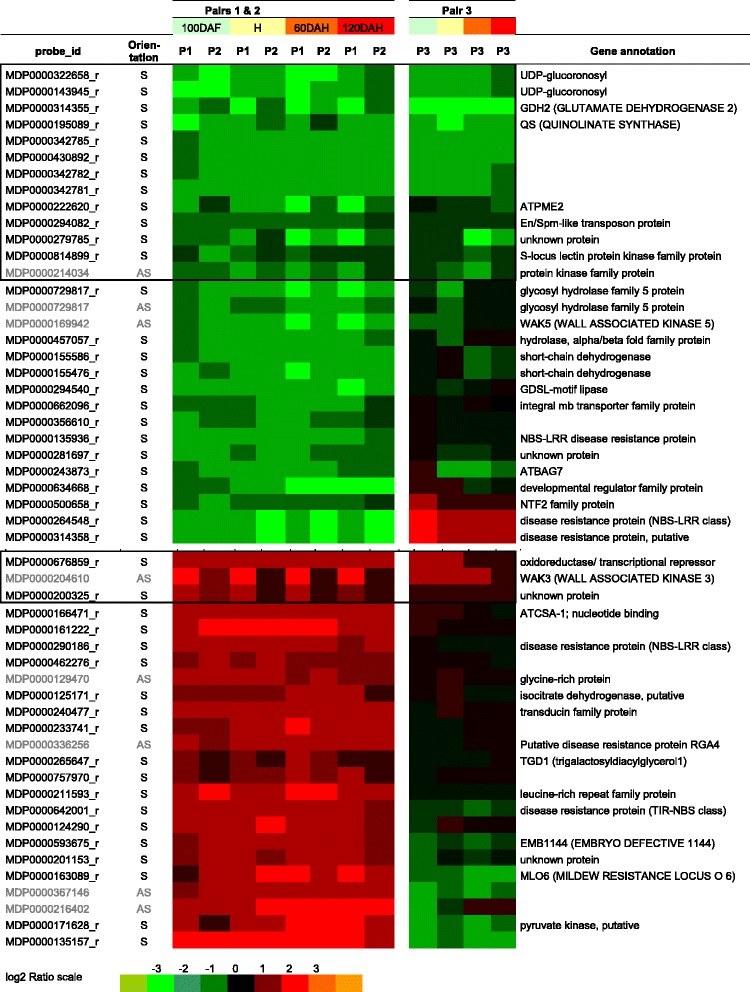
**Selection of differentially expressed genes involved in apple mealiness.** 53 differentially expressed genes identified as displaying consistent profiles for the pairs M40/M49 (P1) and M74/M20 (P2) during all apple maturation are shown here. The pair M48/M16 (P3) was used to validate their expression patterns and to select few relevant genes (encircled by squares). Orientation indicates the orientation of the transcribed mRNA strand (S: sense; AS: antisense). The color scale (below) corresponds to log2 ratios between mealy versus non-mealy parenchyma transcript expression values (Red refers to up-regulated genes in mealy, green in non-mealy hybrids). The short gene annotations are based of Arabidopsis gene homologies.

The majority of phenotypic changes associated with mealiness were observed 60 days after harvest. However, it is likely that molecular events are initiated early in the kinetic of fruit development. Based on this hypothesis and in order to identify genes possibly useful as early molecular markers of mealiness, the study was focused on genes which displayed differential expression patterns during the entire apple fruit development and cold storage period. Using these criteria, 53 genes were selected (Figure 3B). Apples from the M48 hybrid were always less mealy than apples from M40 and M74 (Table 1). Therefore, the third comparison between M48 and M16 apples was used to select genes involved in the development of fruit mealiness regardless of the severity of the phenotype. Genes with two distinct profiles were selected. This drastic selection led to the identification of 16 relevant genes (Figure 4). Thirteen genes were down-regulated at all time points for all 3 pairs such as MDP0000322658, similar to AT5G49690 from *Arabidopsis thaliana* coding for a glycosyltransferase (GT) potentially involved in pectin synthesis, and MDP0000222620, similar to AT1G53830 coding for a pectin methylesterase (PME) involved in pectin modification. Three transcripts were up-regulated including the AS transcript of the gene MDP0000204610 similar to AT1G21240 annotated as “Wall Associated Kinase” (WAK). WAKs are pectin receptor-like proteins, and some Arabidopsis WAKs have been shown to be involved in cell expansion during plant development [25,26]. Apples are climacteric fruits and hormones, particularly ethylene, are known to play a central role during fruit maturation [27]. In this study, no genes related to hormonal pathways were differentially expressed during the complete time course. Such results are not surprising as early cellular events during ripening are hypothesized to be less dependent on ethylene [28,29]. This strict selection can also explain why hormone-related genes are not revealed. However, ethylene production was measured during storage and no significant difference was noticed between mealy and non-mealy apples at all the time points (see protocol and results In Additional file 4). Some genes displayed differential expression patterns at least at one time point, but none of them were validated using the third pair. This result does not question the role of hormonal pathways during apple ripening but it suggests that a hormonal pathway may not be the key regulator of mealiness in the studied population. Moreover, fruit ripening results from both ethylene-dependent and ethylene-independent pathways [10,11,30]. These results support the hypothesis that mealiness and softening may involve two different mechanisms.

**Figure 5:**
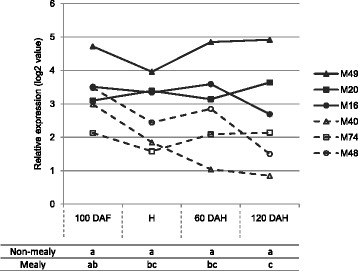
**Expression patterns of** ***MdPME2*** **for each individual hybrid as determined by microarray analysis at four developmental stages (100 DAF, H, 60 DAH and 120 DAH).** Hybrids displayed two different expression patterns: high and stable PME gene expression in non-mealy hybrids (bold lines) contrary to lowest and decreasing gene expression in mealy hybrids (dash lines). The transcript levels were normalized with the Lowess method. Normalized intensities (i.e. expression levels) were then subtracted from the background. Table refers to Kruskall-Wallis results between mealy and non-mealy hybrids at each time point.

These 53 selected genes are potentially involved in mealiness development during apple fruit ripening and cold storage. Among them, the MDP0000222620 gene coding for a protein annotated as a PME is one of the most relevant as PMEs have major role in pectin remodeling in plant cell wall. PMEs are known to be involved either in the stiffening or in the loosening of the cell wall [31]. In the case of fleshy fruits, the role of PMEs is usually associated with pectin degradation that occurs during fruit ripening [32]. That is in accordance with the observation that parenchyma cells from mealy fruit tend to easily detached from each other due to the alteration of the pectin rich middle lamina. In the present study, MDP0000222620 transcript expression levels were significantly higher in non-mealy than in mealy fruit during the whole kinetic of development (Figure 5), suggesting that this gene might be involved in cell wall stiffening. Furthermore, the expression level of MDP0000222620 was higher in M48 (the least mealy hydrid) compared to M40 and M74 hybrids (Figure 5). MDP0000222620 transcript expression pattern was also consistent with phenotypic variations.

### *MdPME2* cloning and the apple PME family

**Figure 6:**
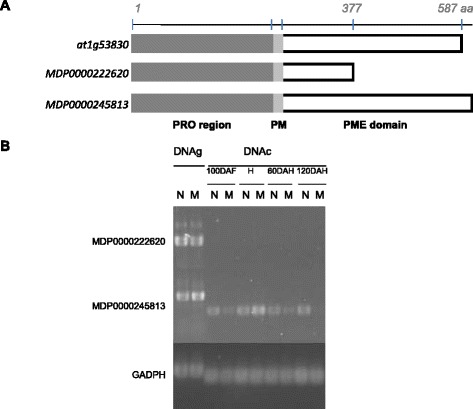
***MdPME2*** **cloning and validation by semi-quantitative RT-PCR. A** Analysis of MDP0000222620 sequence annotated on *Malus x domestica* genome [22]. *At1g53830* is the *Arabidopsis thaliana* most similar sequence. MDP0000245813 is the complete sequence identified from the 3’ RACE analysis. All 3 protein sequences are characterized by the presence of a PRO domain region (PF04043) followed by a putative processing motif [33,69]. The PME domain (PF01095) of MDP0000222620 is incomplete. **B** Confirmation of the cloned gene expression profiles with 2 PME specific primer sets by semi-quantitative RT-PCR for 30 cycles. M20 genomic DNA (DNAg) was used as positive control. Genes expression were studied for pair 2 (M20/M74) during complete fruit kinetic. MDP0000245813 could be amplified with clear difference between mealy (M) and non-mealy (N) genotypes, while there was no amplification with MDP0000222620, and GADPH expression remained at a relatively constant level.

Even if the probe AryANE_v1_00084532 was firstly assigned to the MDP0000222620 gene, we actually showed that it reveals a close homolog of it: MDP0000245813. The complete CDS sequence, hereinafter named *MdPME2*, was cloned from M20 apples and was shown to share 97% of similarities to the MDP0000245813 gene identified on the alternative genes set published [22] (Figure 6A, sequences alignments in Additional file 5). The probe sequence corresponding to MDP0000222620 is only 80% similar to MDP0000245813 sequence. Semi quantitative RT-PCR clearly showed that only the MDP0000245813 gene and not the MDP0000222620 gene was expressed in both M20 and M74 genotypes (Figure 6B). Therefore, the probe designed on MDP0000222620 sequence actually reveals the transcriptional activity of the MDP0000245813 gene.

*In silico* analysis of the MDP0000245813 protein sequence, confirmed the presence of a processed amino-terminal PRO domain similar to a PME inhibitor domain (PF04043) followed by a complete catalytic PME domain (PF01095). A processing motif (RRLL) was also identified between both domains (Figure 6A). This latter was described as a cleavage site suggesting the cleavage of the PRO domain during the process of the protein maturation [31,33]. The current knowledge on the PME family and these observations support the hypothesis that *MdPME2* codes for a pectin methylesterase.

In all studied species, genes coding for pectin methylesterases belong to large multigenic families and their members might be involved in different developmental processes [33,34]. To determinate if other *MdPME* genes are involved in development of fruit mealiness, all apple *MdPME* homologues were identified within the apple v1 genome sequence [22].

Based on protein similarities to the PME conserved domain (PF01095), 60 genes were identified as potential pectinesterase family members. Molecular phylogenetic analysis showed that the *MdPME* family is divided in two groups. As observed with the Arabidopsis *AtPME* familly [33], PME proteins from group type I are characterized by the presence of a PRO domain which is absent in PMEs from group type II. The *MdPME2* belonged to the type I characterized by PME proteins displaying a PRO domains (PF04043) (Additional file 6).

**Figure 7:**
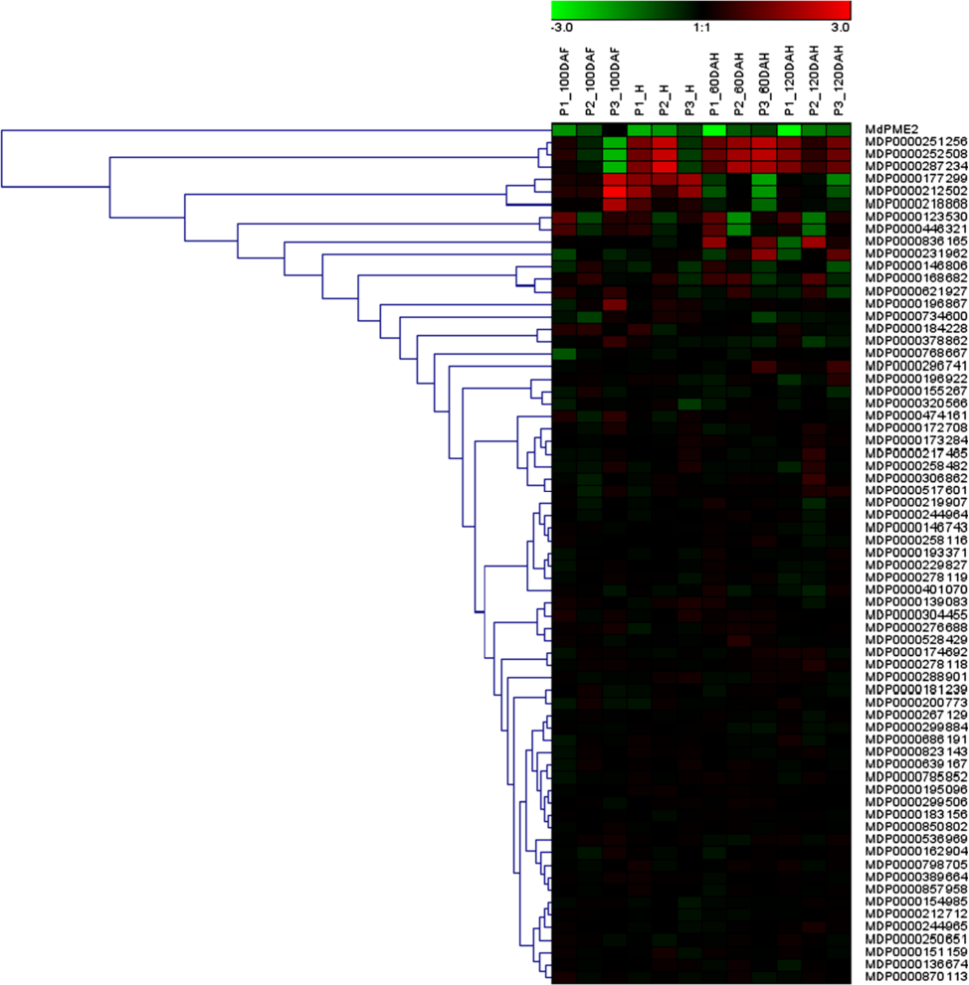
**Hierarchical cluster analysis of the 60 PMEs genes expressions in both 3 pairs of hybrids during complete fruit kinetic using Genesis software.** Each row represents one PME gene. P1, P2 and P3 correspond to pairs M40/M49, M74/M20 and M48/M16 respectively. The *MdPME2* gene only displayed an expression profile consistent with mealiness development.

Moreover, *MdPME2* displayed a unique differential expression pattern during fruit maturation among all *MdPMEs* annotated genes (Figure 7). Few PMEs annotated genes displayed differential expression patterns between mealy and non-mealy apples, but none of them remained consistently differentially expressed during the kinetic of development. Roles of other PMEs in cell wall remodelling during apple maturation is not excluded. However, *MdPME2* is the only gene of the family to be more highly expressed during the whole kinetic analysis in non-mealy hybrids compared with mealy apples. This result underlined the specific role of *MdPME2* in apple mealiness.

### MdPME2 subcellular localization

**Figure 8:**
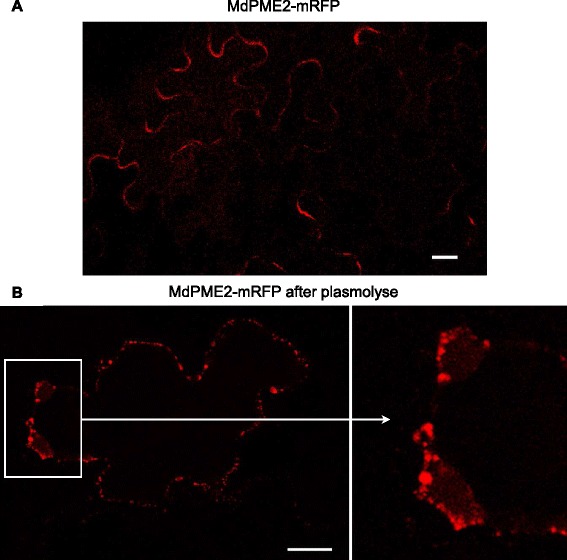
**Subcellular localisation of the** ***MdPME2*** **-RFP fusion protein transiently expressed in** ***Nicotiana tabacum*** **leaves.** The fluorescence of the 35S-MdPME2-mRFP expressed in *N. tabacum* cells construct was visualized using a confocal laser scanning microscope. The cell wall localization was monitored before and after cell plasmolysis confirming the fused protein exportation into the apoplasm. Scale bars = 20 μm.

To investigate the subcellular localization of the MdPME2, a C-terminal translational mRFP fusion was generated and transiently expressed in *N. tabacum* leaves. The mRFP signal was predominantly observed at the cell periphery, consistent with expected cell wall localization (Figure 8A). To further confirm the cell wall localization, transformed leaves cells were plasmolysed. As expected, the dehydration and compression of the cytoplasm induced dissociation of the plasma membrane from the cell wall. The mRFP signal was observed both in the apoplasm and in the cell wall confirming the exportation of the recombinant protein (Figure 8B). This subcellular localization is in accordance with the possible role of the *MdPME2* in the modification of the cell wall structure in mealy apples, particularly the pectin components.

### Apple fruit pectin characterization

To investigate whether pectin was altered during ripening of apple mealy apples the degree of methylesterification (DM) of uronic acids was determined on alcohol insoluble materials (AIMs) from pairs 1 and 2 in 2009 and 2010.The DM was about 75% at 100DAH. It decreased slowly until harvest and remained stable during cold storage (Additional file 7). Hence, these results indicated, as expected, that the apples underwent a de-methylation process through apple development before harvest and in early stage of ripening but they did not reveal global differences between mealy and non-mealy pectins when analyzed at the whole fruit level.

Another approach was to investigate changes in apple pectin architecture. The fine structure of homogalacturonans (HGs) was determined using pectin enzymatic oligosaccharide fingerprinting with oligouronides mass profiling. The peak areas of individual ion signals of the mass spectra were used to calculate relative abundance of each structure allowing the identification of different patterns of methylation and acetylation on the oligouronides. A direct comparison of each oligouronide using Kruskal-Wallis tests showed significant differences between mealy and non-mealy apples. Mealy apples displayed a significantly higher abundance of highly methylesterified structures (DU4m2, DU4m3, DU5m3 and DU5m4) during almost the complete kinetic. Non-mealy apples displayed higher abundance of highly methylesterified structures which are acetylated, such as DU5m4a1 or DU6m2a2, mainly from 60DAH onwards (Additional file 8). These results are consistent with *MdPME2* expression which is higher in non-mealy genotypes resulting in de-methylesterified blocks of free carboxyl groups. An abundance of highly methylesterified but acetylated moieties were also measured in non-mealy apples. O-acetylations of pectins may reduce pectin accessibility to PME enzymes [35]. Therefore PMEs may not act on these acetylated blocks. These results suggest that PMEs demethylesterify pectin in a processive manner leading to demethylesterified blocks. Some local structural rearrangements may explain some specific cell wall functionalities. Furthermore, a putative pectin acetylesterase, MDP0000162976, was significantly less expressed in non-mealy hybrids (pairs 1 and 2) at 120DAH (Additional file 2). The role of a pectin acetylesterase is to cleave the ester bond between a glycosyl carbon and an acetyl group. This enzyme could deacetylated pectins of mealy apples which explains abundance of methyl but not acetylated structures. Its low activity in non-mealy results in maintain of acetylated pectins which cannot be demethylesterified by PMEs. This gene might play an interesting role in regulating PMEs activities and targeting localized demethylesterification of pectins. But it could not be considered as an early molecular marker of mealiness.

## Discussion

### A novel test to quantify mealiness

The confusion made between softening and mealiness results from the lack of easy and objective tests to assess mealiness. Currently, softening is evaluated using a penetrometer by measuring the puncture force required to penetrate the apple flesh using a convex probe [12,36]. However, apple fruits may sometimes soften and in addition develop unfavourable traits such as mealiness [7]. Mealiness has been assessed by measuring the tensile strength required to separate flesh cells [5,37,38] or with sensory analysis performed by a trained panel [11,39]. In the present study, apples were assessed by sensory analysis which remains the most reliable method to evaluate mealiness [40,41]. Indeed, mealiness revealed by sensory analysis was not revealed by penetrometry: no difference between mealy and non-mealy apples was detected based on puncture force. Nevertheless, the softening rate was higher in mealy apples compared with non-mealy apples when assessed with penetrometry. These observations suggest that the rate of softening, rather than the absolute softness of the fruit, may be positively correlated with mealiness.

Sensory analysis by a trained panel of four people is time consuming, expensive, difficult to transpose easily to other laboratories and the number of samples that can be analysed is limited. A few reports investigated some assumptions about cell wall modifications and apple fruit mealiness in order to develop a new test to quantify mealiness. Assumptions are based mainly on microscopic observations of the fracture surface obtained via a tensile test. Different modes of tissue failure can be observed such as cell fracture or cell-to-cell separation. Mealy apples are characterized by neighbouring cells separating from each other at the level of the middle lamella without any damage [5]. SEM observations support this first description of mealiness: cell walls appearing thicker and middle lamella stretching in mealy apples. Mealiness may result from the separation of rounded cells during mastication which leads to a lack of juice and a sandy consistency. The development of the cell cohesion test to assess mealiness was based on this hypothesis. A similar test based on the measurement of the degree of cell separation by shaking flesh tissue disks in a sucrose solution for 8 hours was proposed [42]. In this test, mealiness assessment is based on the weight lost by the disks after shaking. However, cell to cell separation may be the result of individual cell isolation or of clusters of cells. This may lead to imprecision if considering that detachment of clusters of cells is due to an earlier stage of cell wall disassembly than detachment of individual cells.

A link between the observation of angular cells and flesh firmness has been established [43]. However the rapid method used to study cell morphology was based on fruit cell isolation and solubilization of pectin. This method is not appropriate for the characterisation of mealiness. In the present study, the cell cohesion test is a simple test able to discriminate mealy and non-mealy apple fruits within a very short time by quantifying only isolated cells that separated after shaking flesh tissue in water. This test requires little specific material and can be applied to multiple samples.

### Molecular characterization of apple mealiness

Mealiness has often been associated with a defect in the adhesion and rigidity of the cell walls [5,44]. These phenotypes might be the consequence of cell wall disorganization carried out by several parietal enzymes [9,45]. However, only few genes coding for cell wall related proteins were identified in the present transcriptional study. Among these genes, several transcripts displaying unexpected profiles were identified. First, a gene coding for a PME was less expressed in mealy apples although PMEs are known to be involved in pectin solubilization [33]. Secondly, AS expression from a WAK annotated locus was higher in mealy apples. Genes transcripts in AS orientation, often described as precursors of nat-AS-siRNAs, could play a role in post-transcriptional gene silencing of the complementary mRNAs [46]. WAK encoding genes are a small multigenic family. WAKs are supposed to act as signalling proteins that are trans-membrane receptors of pectin residues involved in plant defense responses [25]. It is possible that they are involved in the maintenance of cell wall integrity [47]. Interestingly, this result suggests that WAK AS transcripts might indirectly play a role in apple cell wall integrity, resulting in stiffening of the cell wall. In contrast, another AS WAK transcript was less expressed in mealy apples, but its expression pattern was less consistent amongst the genotypes.

Surprisingly, other genes would have been expected to be differentially expressed but they did not show differential patterns of expression between mealy and non-mealy apples. For example, polygalacturonases (PGs) which function is to depolymerise homogalacturonans by cleaving stretches of unesterified uronic acids (GalA) residues are believed to have a role in apple fruit softening. In [14], the silencing of *MdPG1* in “Royal Gala” resulted in firmer fruits. Further analyses by [29] shown that PGs are mainly involved in the advanced stage of apple softening. In our study, transcripts with sequence similarity to PGs did not display differential expression pattern between mealy and non-mealy apples during the whole time course. However, *Md-PG1* (MDP0000326734) was differentially expressed for both pairs only at 120DAH (Additional file 2) which confirm a role of *Md-PG1* in the advanced stages of mealiness development.

Another gene, *MdAF3*, encoding an α-L-arabinofuranosidase, has been suggested to be associated with mealiness in apple fruits [11]. The authors report distinct patterns of *MdAF3* transcription between certain mealy and non-mealy apple fruits at harvest and after two months of cold storage. *MdAF3* expression did not show a consistent profile in the three analysed pairs in this study, suggesting *MdAF3* is not involved in the development of mealiness.

### Specific role of a *MdPME2* gene in mealiness development

One *MdPME* gene, named *MdPME2*, was identified as differentially expressed in all of the stages of fruit development in this study. *MdPME2* expression remained decreased in mealy apples but remained high in non-mealy apples. *MdPME2* expression was also affected by the fruit production year.

The majority of studies of the role of PMEs in apple fruit ripening has involved measurements of PME activities from fruit set to over ripe stages in “Mondial Gala” [34] or during cold storage of “Fuji” and “Golden Delicious” fruits, classified respectively as firm and soft cultivars [12]. None of these cultivars have ever been classified as mealy. No link between the enzyme activity and *MdPME* mRNA expression was demonstrated. In these studies, two different *MdPME* genes were identified. First, *MdPME1*, which was shown to be specifically expressed in flowers and not in apple fruits [48]. A *MdPME* primer set was used by Wei *et al.* to detect a second *MdPME* gene [12]. In the present work, the gene MDP0000196867 possibly corresponding to the Wei *et al*. primers, was not differentially expressed according to mealiness.

*In silico* analysis of the *MdPME2* protein sequence revealed the presence of a transmembrane domain (signal anchor) required for protein exportation to the cell wall. Localization of the *Md*PME2 protein by mRFP-tagged fusion protein experiments confirm its translocation into the apoplasm where the PME enzyme is hypothesised to act on the cell wall components.

Amongst the 60 identified PMEs, *MdPME2* displayed a unique expression pattern suggesting a specific role during apple development. Many studies have focused on the role of PMEs in pectin depolymerisation during fruit ripening [9,10]. However, recent works tend to re-evaluate this role. In apricot, PME activity increased in a firm cultivar while it decreased in the soft cultivar [49]. Anti-sense inhibition of *SlPE1*, a minor PME isoform in tomato fruits, results in fruits that soften faster than controls [50]. Therefore PMEs could be involved in the deconstruction of the cell wall during fruit development and storage by promoting the action of cell wall hydrolases such as PGs which can degrade the de-esterified pectins. PMEs also may have a role in cell wall stiffening. De-esterified pectins interact with bivalent ions Ca^2+^ and are hypothesised to form egg-box structures. This conformation is supposed to limit the access of PGs to the pectin backbone and therefore to prevent pectin degradation [31]. Results from the present work are consistent with this second property. Maintaining high expression of *MdPME2* in fruit flesh would increase Ca^2+^ bound to the cell wall, subsequently maintaining apple flesh integrity and thus preventing the apparition of mealiness.

Moreover, significant differences were observed in the pectin fine structures between mealy and non-mealy apples while no significant changes were noticed in the degree of methylesterification of the AIM. It has been demonstrated that PMEs can de-esterify homogalacturonic acid (HGA) in various ways. They may act in blockwise or random manners creating micro-domains with different properties within the cell walls [51]. In agreement with the observation of calcium rich zones at tricellular junctions or the corner of intercellular spaces, which remain intact after the rest of the middle lamella had been degraded [52], it has been suggested that local calcium linked gels of low-ester pectin could be responsible for intercellular adhesion [45]. This supports the hypothesis that the identified *MdPME2* might locally change the pectin properties in a way that is not observable at a whole fruit scale but only locally. Recently, differences of pectin structure were identified by immunodetection between two apple cultivars during fruit development [53]. ‘Royal Gala’, a soft cultivar, displayed more highly methylesterified HG regions in corners of tricellular junctions compare to ‘Scifresh’, a firm cultivar, which is associated with corners filled with non or low methylesterified pectin. Both results suggest that PMEs could act locally. Thus, *Md*PME2 might play a role in determining cell-to-cell cohesion during apple fruit ripening by regulating calcium binding to the cell wall.

## Conclusions

In this paper, a new and simple method was developed to quantify mealiness and allowed the characterization of genotypes with contrasting phenotypes for this trait. Analysis of differential expression profiles using the microarray method led to the identification of a number of genes that might be involved in mealiness development in apple fruits. Amongst them, the *MdPME2* gene seems to be a relevant molecular marker associated with apple mealiness. Further investigations to specify the role of *MdPME2* in the molecular events related to the development of mealiness would require an enzymatic activity characterization and antibody production.

## Methods

### Plant material and fruit sampling

Six hybrids progenies issued from the “IM” population segregating for mealiness. This population was derived from a cross between X6681 and X6683, two genitors from the INRA breeding programme (Angers) (Pedigree is available in Additional file 9). The cross was developed in the framework of the European research project High-Quality Disease-Resistant Apple for a Sustainable Agriculture (HIDRAS) [54]. Hybrids were selected for their clear-cut segregation for fruit mealiness, based on sensory analyses, hereinafter named M40, M74 and M48 (mealy), and M49, M20 and M16 (non-mealy). Apples were collected at 100 days after flowering (100DAF), at harvest (H), at 60 and 120 days after harvest (60 and 120DAH) and cold stored at 1°C. Harvest criterions were the optimum maturity based on taste, ground color and starch index of 6–7 [55]. Stored apples were warmed overnight at room temperature prior to analysis. At harvest and storage stages, two apples were submitted to sensory analysis. Flesh tissues of four peeled apples were frozen in liquid nitrogen and stored at −80°C for RNA extraction and enzymatic profiling during four consecutive years (2007, 2009 to 2011). The cell cohesion test was developed and performed using fruits from 10 apple genotypes harvested in 2012 in experimental orchards at INRA, Angers following the same harvest and storage procedure as described above (Additional file 1).

### Sensory and instrumental texture analyses

Sensory analyses were performed by an expert panel of four judges working in pairs as described in [11]. Sensory attributes were mealiness, firmness, crunchiness, juiciness, meltiness and granularity. They were rated on a 9 point scale from 1 (low) to 5 (very high). An apple was considered as mealy if its mealiness score was above 2.5. In addition, instrumental firmness was assessed using an automate penetrometer (TA.XT.-PLUS, Stable Micro System) equipped with a 4 mm-diameter convex probe as described by [56]. Assessments were performed on the two unpeeled and opposite sides of each fruit in the blush and shaded regions. Ten apples were analysed for individual genotype from harvest to 120DAH. Forces in Newtons (N) measured at 7 and 9 mm of displacement were used to estimate an average force Ffmoy (N) [57].

### Cryo-scanning electron microscopy

Apple flesh was ruptured at room temperature and prepared to expose the inner surface of the broken sample for cryo-scanning electron microscopy (SEM). The sample was reduced to a square of 5 mm with a thickness of 2 mm. The fractured biological surface was fixed with carbon on the stub and immediately introduced into the SEM (Phenom G2 Pro, PhenomWorld) in a frozen chamber (−20°C) for 30 min.

### Cell cohesion test

The expert panel scored 158 apples (Additional file 1). The apples were used to develop the cell cohesion test. Three radial cylinders with diameter and thickness of 1 cm each were cut from fresh apple parenchyma. Samples were suspended into 5 ml of tap water in a 50 mL tube, mixed using a vortex at 3000 rpm for 2 min and stained with 50% toluidine blue dye. 40 μl of suspended cells were immediately spotted onto a microscopy slide and visualized with a binocular microscope at 7x magnification (Olympus SZX16). Images were captured with a digital camera. Two replicates per sample were analysed. Automatic counting of individual cells was achieved using the function “*Analyze particles*” from ImageJ software [58] after image conversion from RGB coloured picture to black and white. Statistical analyses were performed with R software. Pearson’s correlation was calculated between individual cell density in the solution and the mealiness score from sensory evaluation and validated with a significance test.

### AIM extraction

Alcohol Insoluble Materials (AIMs) were extracted using an automated extraction method with accelerated solvent extraction unit ASE® 350 (DIONEX, CA, USA). Approximately 4 g of frozen apple fleshes were lyophilized, dried at 40°C overnight under vacuum over P_2_O_5_ and ground into fine powder using FastPrep-24 instrument (MP Biochemicals, CA) at a speed of 6.5 m.s^−1^ for 60 s. Samples were extracted using 80% ethanol at flow 2 mL/min in 22 ml cells of the ASE 350. ASE extractions were performed at 100°C with flow time of 6 min, followed by rinse volume 150%, and a purge time (N_2_) of 30 s. 100DAF samples were extracted twice and H, 60 and 120 DAH samples were extracted three times as technical replicates. The procedure of extraction was conducted for two consecutive years (2009 and 2010). AIMs were dehydrated at 40°C overnight under vacuum over P_2_O_5_. All biochemical measurements were performed from dry AIM.

### Pectin enzymatic profiling

For each AIM sample, 5 mg were suspended in 1 mL of acetate buffer (5 mM, pH 5) and degraded by pectin lyase (0.55 nkatal) prepared according to [59]. Enzymatic digestion and mass spectrometry acquisition were performed according to [60]. Briefly, oligosaccharides in the hydrolysates were analysed by MALDI-TOF MS using an Autoflex III MALDI-TOF/TOF spectrometer (Bruker Daltonics, Bremen, Germany). Spectra were recorded in the mass range m/z 600–1400 and exported to Flex Analysis 3.0 software (Bruker) and preprocessed. Ion masses and intensities were normalized according to the ion peak attributed to DU4m4. Oligosaccharide nomenclature was as follows: the letter U corresponds to uronic acid, the following number refers to the number of residues in the oligomer (i.e., DP degree of polymerization), acetyl and methyl esters substitutions were referred to as a and m, respectively, followed by the amount of groups. According to this nomenclature, DU4m4 refers to an oligo-hexouronide of DP4 fully methyl esterified and unsaturated at the nonreducing end. Each AIM sample was analysed in triplicate. Kruskal-Wallis (p-value < 0.05) analyses were performed with R software on MALDI-TOF MS ion intensities.

### GalA content and degree of methylesterification

GalA content was quantified after sulphuric acid hydrolysis. AIM samples were dispersed in 13 M sulphuric acid for 30 min at 30°C and then hydrolysed in 1 M sulphuric acid (2 h, 100°C). Uronic acids from the acid hydrolysates were quantified using the metahydroxydiphenyl colorimetric acid method.

Analysis of pectin degree of methylesterification (DM) was performed using gas chromatography method after saponification of the AIM. AIM were precipitated after addition of acetone and centrifugation at 14 000 rpm for 10 min and supernatants were transferred into GC vials. A 73-75% DM citrus pectin sample was used as control. Methanol at 0.8 mg mL^−1^ was used as external standard. Butanol at 0.35 mg mL^−1^ was added to each sample as internal standard. GC was performed using a column Optima Wax 30 m × 0.25 mm × 0.25 μm, and a temperature program as following: from 50°C (held for 2 min) to 100°C at 15°C min^−1^, then from 100°C to 217°C (held for 2 min) at 20°C min^−1^. The DM was calculated as moles of methanol per mol of GalA.

### RNA extraction, amplification and microarray hybridization

Total RNAs were extracted from 4 g of frozen fruit flesh tissue ground in liquid nitrogen using a CTAB extraction buffer as described in [11]. mRNAs were amplified, labelled and co-hybridized according to [23] as following: aRNAs were produced with Message AmpII aRNA amplification kit (Ambion) from 200 ng of total RNA. Then, 5 μg of each aRNAs were retrotranscribed and labelled with either Cyanine-3 or Cyanine-5 fluorescent dye (Interchim, Montluçon, France). Labelled samples were combined as 30 pmol for each dye and co-hybridized to the Nimblegen microarray AryANE v1.0 containing 135000 60-mers oligonucleotide probes as described in [23]. Deva software (Nimblegen) was used to extract pair-data files from the scanned images, obtained using the MS200 microarray scanner (Roche Nimblegen).

Based on sensory score, genotypes were associated in 3 mealy/non-mealy pairs: P1 (M40/M49), P2 (M74/M20) and P3 (M48/M16). The reference RNA, a non-mealy hybrid, was hybridized along with the treatment RNA, a mealy hybrid, with the opposite dye. Expression for each gene was worked out as the mean and standard error of two biological replicates hybridized on two independent arrays with fluorochrome reversal (dye switch: 2009 and 2010) for the four time points and for the three pairs, for a total of 24 arrays. P1 and P2 were also analysed at harvest and 60 DAH for 2007 and 2011 (6 arrays).

### Microarray analyses

All statistical analyses were conducted as described [61] with the R software (R development Core Team, 2009). Briefly, data were normalized with the Lowess method, and differential expression analyses were carried out using the lmFit function and the Bayes moderated t test using the R package LIMMA [62] from the Bioconductor project. To estimate gene expression levels, the normalized expression values were corrected from background.

Genes were considered differentially expressed if the t-test *P-*values of the paired sample were below 1%. Genesis software (http://genome.tugraz.at/genesisclient/genesisclient_description.shtml) was used to visualize results. Functional classification was based on Mapman ontology [63]. An enrichment analysis (Wilcoxon rank sum test) was performed with MapMan software. Kruskal-Wallis (p-value < 0.05) analyses were performed on *MdPME2* intensities with R software.

The microarray data have been submitted to the Gene Expression Omnibus data base (http://www.ncbi.nlm.nih.gov/geo/) under the accession number GSE59947.

### RT-qPCR and semi-quantitative PCR

Total-RNA samples used for the microarrays experiments were treated with 2U of DNAse I (Promega, USA). cDNAs were synthesized using 1 μg of DNA-free-RNA with 1 μl of oligo(dT)15 (Promega), 200U of MMLV-RT (Promega) and 0.5 mM of each dNTP for 1 hr at 42°C in 25 μl. The qPCR were performed in duplicate with 3 μl of 10x diluted cDNA in a final volume of 15 μl using the IQ SYBR Green Super Mix (Quanta biosciences). Specific forward and reverse primers concentration was set at 0.3 μM. qPCR reactions were run on a CFX96 Touch™ Real-Time PCR detection system (Bio-Rad, USA) under the following conditions: an initial step at 95°C for 3 min, then 40 cycles of 95°C for 10 sec and 60°C for 1 min, and a final dissociation curve ranging from 60°C to 95°C. Amplification and dissociation curves were monitored and analysed with CFX Manager software v1.6 (Bio-Rad). Primers were designed for short and specific amplification of the microarray probe region of the selected sequences with Primer 3 plus software (http://www.bioinformatics.nl/cgi-bin/primer3plus/primer3plus.cgi) (Additional file 10). For each run, single product amplification was confirmed by melt curve analysis. The amplification efficiency was tested for each primer pairs using a dilution curve method over a 6 point dilution series (from 0.1 to 0.002) on a pool of cDNAs containing all genotypes and developmental stages. Primer pairs selected for further analysis have efficiencies above 91%. RT-qPCR was carried out for 5 genes, 2 pairs at different time points.

Based on the microarray results, three reference genes with similar expression levels in all samples were selected to calculate a normalization factor: MDP0000645828 (GADPH), MDP0000217860 and MDP0000271281 (respectively annotated as drought-responsive family protein and putative porin). Relative expression level was calculated using a formula derived from the 2^–ΔΔCt^ method [ΔCt = (Ct_tag_–Ct_ref_)], where Ct is the threshold cycle, tag is the target gene, and ref is the reference gene [64]. Specifics primers were designed for MDP0000222620 and MDP0000245813 gene based on GDR CDS sequences (http://www.rosaceae.org/) (Additional file 10).

Semi-quantitative PCR were carried out on cDNA from M20 and M74 at four developmental stages, each one being a pool of 2009 and 2010 cDNAs. Reactions were run for 30 cycles on diluted cDNA (3 point dilution series from 0.1 to 0.001) using GoTaq®Flexi DNA polymerase (Promega) according to the manufacturer’s protocol.

### Genome-wide identification and analysis of PME genes in *Malus domestica* genome

The HMM profile of the Pectinesterase proteins (PF01095) was downloaded from the Pfam database (http://pfam.sanger.ac.uk), and used as a query to search the translated Apple genome (http://www.rosaceae.org/) using the HMMER 3.0 program with default parameters (hmmer.org). All sequences collected were checked using the NCBI conserved domain database [65]. Protein coding genes without a complete pectinesterase domain (EC 3.1.1.11) or with extra and incoherent domain were discarded from further analyses.

Multiple protein sequence alignments were performed with the online MUSCLE tool (http://www.ebi.ac.uk/Tools/msa/muscle/). Phylogenetic analysis was carried out by the neighbor-joining method using MEGA 5 software [66] using a p-distance model and pairwise deletion for missing data treatment; 1,000 replicates were used for bootstrap analysis.

### *MdPME2* full length cDNA cloning

A 3’RACE cDNA amplification was performed with the 3’RACE system kit (Invitrogen) on 1 μg of DNAse free total-RNA from genotype M20 according to the manufacturer’s protocol. A forward primer (F6) was designed on the AryANE_v1_00084532 microarray probe sequence. The PCR program included an initial denaturation at 94°C for 5 min, then 40 cycles of 94°C for 30 sec, 60°C for 45 sec and 72°C for 1 min 30 sec, and a final extension at 72°C for 10 min. A reverse primer (R) was then designed on the obtained sequence.

Full length cDNA was cloned using the high fidelity Phusion DNA polymerase (Finnzymes) and primers MDP222620 F5 and R on a pool of cDNA from the M20 genotype according to the manufacturer’s instructions. A nested PCR was then performed on the first PCR product with the forward and reverse primers MDP222620-F1 and R. The PCR product was purified using the PCR clean-up system (Promega) according to the manufacturer’s instructions, and cloned in the pENTR/D-TOPO vector using the pENTR directional TOPO cloning kit (Invitrogen, USA) according to the manufacturer’s instructions. One Shot TOP10 chemically competent *E.coli* cells (Invitrogen) were then transformed by heat shock with 2 μL of the reaction. The transformed bacteria were grown overnight at 37°C on LB agar medium containing 50 μg/ml kanamycin as selective agent. Positive clones were selected by PCR amplification of the inserted cDNA using F and R primers and a plasmid sequenced to confirm the proper orientation of the cloned fragments.

All DNA sequencing were performed by Genoscreen (http://www.genoscreen.fr) and *MdPME2* sequence was submitted to [GenBank:KM252690]. All primers are described in Additional file 10.

### Subcellular localization by transient expression in *Nicotiana tabacum*

The full length *MdPME2* cDNA was cloned in the destination vector pH7RWG2 (containing mRFP and allowing a C-terminal fusion to *MdPME2*, http://gateway.psb.ugent.be/) by recombination using the gateway cloning system (Life Technologies) according to the manufacturer’s instructions. Plasmids were extracted using Nucleospin plasmid kit (Macherey Nagel) according to the manufacturer’s protocol.

Plasmid containing the MdPME2-mRFP construct was introduced into *Agrobacterium tumefaciens* strain C58C1 by electroporation. The transformed *A.tumefaciens* were inoculated into 2 ml of LB supplemented with 50 μg/ml of rifampycine, 25 μg/ml of gentamycine and 50 μg/ml of spectinomycin overnight at 28°C. The diluted bacteria were used to infiltrate young *Nicotiana tabacum* leaves according to the protocol previously described [67]. The sub-cellular localization was analyzed 2 to 3 days after infiltration using a confocal laser scanning microscope (Nikon A1, BIBs platform at INRA Nantes). To verify cell wall localization, some leaf fragments were plasmolysed for 5 min in 30% glycerol prior to microscopic observation.

### Availability of supporting data

The nucleotide sequence supporting the results of this article is available in the GenBank repository (http://www.ncbi.nlm.nih.gov/genbank/) under the identifier GenBank:KM252690. The microarray data are available on Gene Expression Omnibus [68] database (http://www.ncbi.nlm.nih.gov/geo/) under the accession number GSE59947. MdPMEs sequence alignment and phylogenic tree data are available on Dryad (http://datadryad.org/) under the DOI number: 10.5061/dryad.4337n.

## Additional files

Additional file 1:
**Results of cell cohesion test.** S1.A Mealiness score and results of the cell cohesion test (number of cell per μl). 158 apples of 10 different genotypes were used to develop this new test in 2012. S1.B Dispersion graph of the correlation between mealiness and cell cohesion test results. The y-axis represents the cell cohesion test results expressed as the number of isolated cells counted by μl. The x-axis represents sensorial mealiness. Pearson’s correlation coefficient was calculated. Additional file 2:
**Differentially expressed genes of pairs 1 and 2 which displayed similar expression patterns at one or more fruit development stages.** Pairs are M49/M40 and M74/M20. Time points are 100DAF (100 days after flowering), H (harvest), 60 and 120 DAH (respectively 2 and 4 months of cold storage). The significance level was set at p < 0.01. Additional file 3:
**Validation of microarrays results by quantitative real-time PCR (qRT-PCR).** S2.A: Validation of microarrays results by quantitative real-time PCR (qRT-PCR) for selected genes for 2 pairs of genotypes at two time points. Reported ratios were calculated on the basis of normalized data for microarray analyses (log2 ratio) and as normalized expression for qPCR (Ct ratio). H: harvest, DAH: days after harvest. S2.B: Correlation between qRT-PCR ratios and microarray log2 ratios is shown for the same genes, along with Pearson correlation coefficient. Additional file 4:
**Ethylene measurements during storage.**
Additional file 5:
**Sequence alignments.**
Additional file 6:
**Evolutionary relationships between 60 apple PMEs.** The tree is based on the alignment of protein sequences using MUSCLE and it is constructed using the neighbor-joining method and a bootstrap test with 1000 iterations using MEGA5 software. Black lines grouped PME genes of type I, including *MdPME2*, and gray are of type II according to *Arabidopsis thaliana* PME groups [33]. Additional file 7:
**Degree of methylesterification (DM) of apple cell walls (2009, 2010) for mealy (M40, M74) and non-mealy hybrids (M20, M49) during the kinetic.** Values are the means of 3 technical replicates for both years. Error bars represent the standard deviation. Additional file 8:
**Pectin enzymatic profiling results.** S8.A Sum up of significant differences between mealy and non-mealy fruits for each oligouronide during the complete apple fruit cycle according to Kruskall-Wallis tests. The oligouronides were identified from MALDI-TOF MS spectra after pectin-lyase hydrolysis of cell wall preparations. Oligouronides nomenclature is described in the materials and methods. Colors refer to significant differences (p-value < 0.05) between mealy and non-mealy hybrids: dark grey is for a higher abundance in mealy fruits, light grey: a higher abundance in non-mealy fruits. S8.B Bar charts of abundance of each oligouronides for mealy (M40, M74) and non-mealy (M49, M74) hybrids at the four fruit developmental stages obtained from MALDI-TOF MS spectra after pectin-lyase hydrolysis of cell wall preparations. Oligouronides nomenclature is described in the materials and methods. Letters on bar charts indicate groups of significantly different abundance (Kruskall-Wallis test). M: mealy; NM: non-mealy. Additional file 9:
**IM population within HIDRAS pedigree [** 24 **].**
Additional file 10:
**List of primers used for RT-qPCR and cloning.** S10.A Primers used for the RT-qPCR. For each selected gene, the expression pattern obtained by microarray analysis is specified (up or down in mealy fruits compared to non-mealy). Three housekeeping genes (HK) were used to calculate the normalisation factor. S10.B Primers used for *MdPME2* cloning.

## Abbreviations

AIM: Alcohol insoluble material
AS: Anti-sense
DAF: Days after flowering
DAH: Days after harvest
DM: Degree of methylesterification
GalA: Galacturonic acids
H: Harvest
HG: Homogalacturonans
PG: Polygalacturonase
PME: Pectin methylesterase
WAK: Wall Associated Kinase
