# Supplementary material for: Multiscale investigation of mealiness in apple: an atypical role for a pectin methylesterase during fruit maturation
Source: BMC Plant Biol. 2014 Dec 31;14:375. doi: 10.1186/s12870-014-0375-3 (PMC4310206; doi:10.1186/s12870-014-0375-3)

Additional file 3. Validation of microarrays results by quantitative real-time PCR (qRT-PCR)

S2.A: Validation of microarrays results by quantitative real-time PCR (qRT-PCR) for selected genes for 2 pairs of genotypes at two time points. Reported ratios were calculated on the basis of normalized data for microarray analyses (log2 ratio) and as normalized expression for qPCR (Ct ratio). H: harvest, DAH: days after harvest.

stage	Couple	MDP number	Gene annotation	qRT-PCR (Ct Ratio)	Microarray (log2 ratio)
H	M74 / M20	MDP0000222620	AtPME2	-2.12	-1.9
H	M74 / M20	MDP0000322658	UDP-glucoronosyl	-6.17	-2.58
H	M74 / M20	MDP0000393227	FQR1	-3.82	-2.45
H	M74 / M20	MDP0000671440	GDH1	-1.8	-1.07
H	M74 / M20	MDP0000303194	CRK10	8.16	3.17
60 DAH	M74 / M20	MDP0000222620	AtPME2	-2.67	-1.62
60 DAH	M74 / M20	MDP0000393227	FQR1	-0.3	0.06
60 DAH	M74 / M20	MDP0000671440	GDH1	-2.49	-0.85
60 DAH	M74 / M20	MDP0000303194	CRK10	7.24	2.06
60 DAH	M74 / M20	MDP0000322658	UDP-glucoronosyl	-11.03	-2.48
H	M40 / M49	MDP0000322658	UDP-glucoronosyl	-9.37	-2.6
H	M40 / M49	MDP0000393227	FQR1	-3.56	-2.68
H	M40 / M49	MDP0000303194	CRK10	4.12	3.22
60 DAH	M40 / M49	MDP0000222620	AtPME2	-3.34	-3.92
60 DAH	M40 / M49	MDP0000393227	FQR1	-3.73	-2.52
60 DAH	M40 / M49	MDP0000322658	UDP-glucoronosyl	-5.99	-3.26

S2.B: Correlation between qRT-PCR ratios and microarray log2 ratios is shown for the same genes, along with Pearson correlation coefficient.

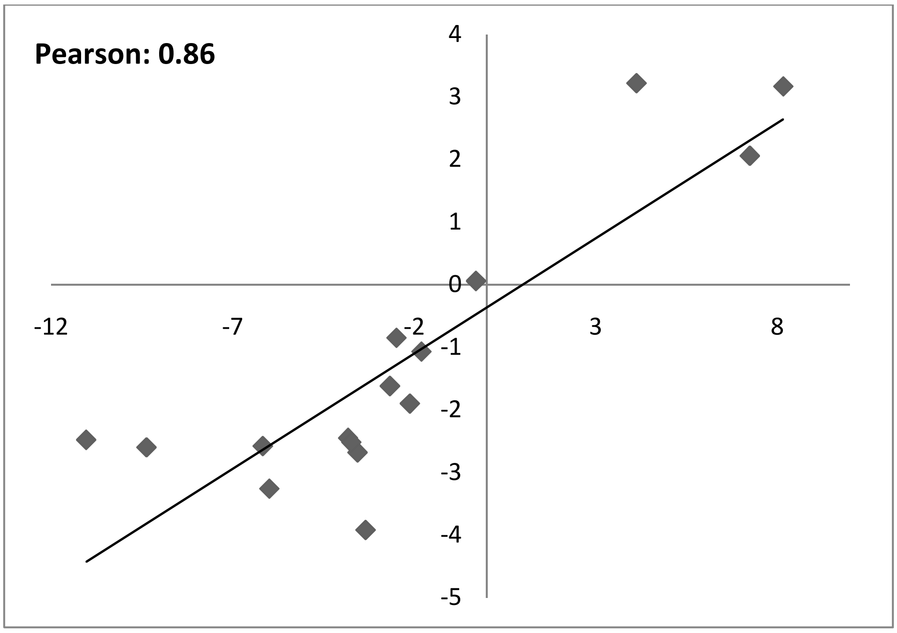

Supplement: Additional file 3: — Validation of microarrays results by quantitative real-time PCR (qRT-PCR). S2.A: Validation of microarrays results by quantitative real-time PCR (qRT-PCR) for selected genes for 2 pairs of genotypes at two time points. Reported ratios were calculated on the basis of normalized data for microarray analyses (log2 ratio) and as normalized expression for qPCR (Ct ratio). H: harvest, DAH: days after harvest. S2.B: Correlation between qRT-PCR ratios and microarray log2 ratios is shown for the same genes, along with Pearson correlation coefficient. [file 12870_2014_375_MOESM3_ESM.pdf]
