# Supplementary material for: Multiscale investigation of mealiness in apple: an atypical role for a pectin methylesterase during fruit maturation
Source: BMC Plant Biol. 2014 Dec 31;14:375. doi: 10.1186/s12870-014-0375-3 (PMC4310206; doi:10.1186/s12870-014-0375-3)
Supplement: Additional file 4: — Ethylene measurements during storage. [file 12870_2014_375_MOESM4_ESM.pdf]

Additional File 4: Ethylene measurements during storage

Materials and Methods:

The fruits were sampled in 2010 and 2011 for ethylene quantification during storage. Ten fruits were transferred to a hermetically closed container and ethylene contents were analysed using an Ethylene Atmosphere Sampling Instrument (EASI-1, Absoger France).

To identify any statistical differences between mealy and non-mealy apples, student tests were performed at each time point.

Results:

Ethylene accumulation in fruits from individual hybrids. The values represent the mean of 10 measurements. Results of student test at each time point between mealy and non-mealy are mentioned below.

		**2010**		**2011**	
Phenotype	**Hybrid**	**H**	**60DAH**	**60DAH**	**120DAH**
NM	M16	0,17	33,92	5,11	44,10
NM	M20	0,88	2,61	-0,64	-1,79
NM	M49	0,65	5,49	0,87	29,71
M	M40	1,94	5,25	0,77	6,81
M	M48	1,41	59,87	27,26	61,12
M	M74	6,89	103,82	31,16	36,14

Results of student test:	0,24	0,38	0,25	0,75
