# Supplementary material for: Multiscale investigation of mealiness in apple: an atypical role for a pectin methylesterase during fruit maturation
Source: BMC Plant Biol. 2014 Dec 31;14:375. doi: 10.1186/s12870-014-0375-3 (PMC4310206; doi:10.1186/s12870-014-0375-3)
Supplement: Additional file 5: — Sequence alignments. [file 12870_2014_375_MOESM5_ESM.pdf]

```
                                           20                    40                    60
MdPME2               ATGGCCAAAA TCAAAGAGTT TTTAGCCAGA ATGTCCGAAT CAGGCAAAAA ATTATCCACA AACAAGAAGA 70
MDP245813_cds_GDR    ATGGCCAACA TCAAAGAGTT TTTAGCCAGA ATGTCCGAAT CAGGCAAAAA ATTATCCACA AACAAGAAGA 70
MDP222620_cds_GDR    ATGGCCAAAA TCAAAGAGTT TTTGACCAAA ATGTCCGAAT CGGGCAAACA AATATCCACA AACAAGAAAC 70
AryANE_v1_00021521   ---------- ---------- ---------- ---------- ---------- ---------- ---------- -

                                80                   100                   120                   140
MdPME2               ACAAGAAGCT CTTCATATCC CTTTTCGCAA CCATCCTACT CGTGGGCGCC GTCATCGGCA TTGTCACCGG 140
MDP245813_cds_GDR    ACAAGAAGCT CTTCATATCC CTTTTCGCAA CCATCCTACT CGTGGGCGCC GTCATCGGCA TTGTCACCGG 140
MDP222620_cds_GDR    ACAAGAAGCT TTTCATATCC CTTTTCGCAA CCATCCTACT AGTCGGCGCC ATCGTCGGCA TTGTCACCGG 140
AryANE_v1_00021521   ---------- ---------- ---------- ---------- ---------- ---------- ---------- -

                                          160                   180                   200
MdPME2               AGTAAAGTCC TCCAAAAACA ACTCCGACGA CGAAACCATC GAGGCCTCCC ACGCCATAGT CAAATCCTCA 210
MDP245813_cds_GDR    AGTAAAGTCC TCCAAAAACA ACTCCGACGA CGAAACCATC GAGGCCTCCC ACGCCATAGT CAAATCCTCA 210
MDP222620_cds_GDR    AGTCAAGTCC TCCAAAAACA ACTCCGACGA CGAAACCAAC GAGGCCTCCC ACGCCATAGT CAAATCCTCA 210
AryANE_v1_00021521   ---------- ---------- ---------- ---------- ---------- ---------- ---------- -

                               220                   240                   260                   280
MdPME2               TGCAGCTCCA CCCTCTACCC TGATCTGTGC TTTTCAACTC TTGCCCTTCA CCCGGAAGCC TCCAAAAAGG 280
MDP245813_cds_GDR    TGCAGCTCCA CCCTCTACCC TGATCTGTGC TTTTCAACTC TTGCCCTTCA CCCGGAAGCC TCCAAAAAGG 280
MDP222620_cds_GDR    TGCAGCTCCA CCCTCTACCC TGAACTGTGC TTTTCCACTC TTGCACTTCA CCCCGAAGCC GCCAAAAAGG 280
AryANE_v1_00021521   ---------- ---------- ---------- ---------- ---------- ---------- ---------- -

                                          300                   320                   340
MdPME2               TTTCGAGCCA GAAGGACGTG ATAGAATTGT CACTGAACAT CACGACCACG GCGGTGCAGC ACATCTTCTT 350
MDP245813_cds_GDR    TTTCGAGCCA GAAGGACGTG ATAGAATTGT CACTGAACAT CACGACCACG GCGGTGCAGC ACATCTTCTT 350
MDP222620_cds_GDR    TTTCGAGCCA GAAGGACGTG ATAGAATTGT CATTGAACAT CACGACCAAG GCGGTTCAGC ACAACTTCTT 350
AryANE_v1_00021521   ---------- ---------- ---------- ---------- ---------- ---------- ---------- -

                               360                   380                   400                   420
MdPME2               CACCGTCGAG AAGCTGCTGA AGTCCAGGAA GAACAAGCTC ACTAAGCGTG AGAAGGGCGC TCTCCACGAC 420
MDP245813_cds_GDR    CACYGTCGAG AAGCTGCTGA AGTCCAGGAA GAACAAGCTC ACTAAGCGTG AGAAGGGCGC TCTCCACGAC 420
MDP222620_cds_GDR    CACTGTCGAG AAGCTGCTGA AGTCCAGGAA GAATAAGCTC ACTAAGCGTG AGAAGGGCGC TCTTCACGAC 420
AryANE_v1_00021521   ---------- ---------- ---------- ---------- ---------- ---------- ---------- -

                                          440                   460                   480
MdPME2               TGTTTGGAGA CCATTGACGA AACCCTCGAC GAGCTCCACG AGGCTGTCGA GGATCTCCAT GAGTACCCCA 490
MDP245813_cds_GDR    TGTTTGGAGA CCATTGACGA AACCCTCGAC GAGCTCCACG AGGCTGTCGA GGATCTCCAT GAGTACCCCA 490
MDP222620_cds_GDR    TGTTTGGAGA CCATTGATGA AACTCTCGAC GAGCTTCACG ATGCTGTGGA GGACCTCCAT GAGTACCCTA 490
AryANE_v1_00021521   ---------- ---------- ---------- ---------- ---------- ---------- ---------- -

                               500                   520                   540                   560
MdPME2               ATAACAAGAC TTTGGTTCAG CATGCTGATG ACCTTAAAAC TTTGCTAAGC TCCGCCATTA CCAACCAAGA 560
MDP245813_cds_GDR    ATAACAAGAC TTTGGTTCAG CATGCTGATG ACCTTAAAAC TTTGMTAAGC TCCGCCATTA CCAACCAAGA 560
MDP222620_cds_GDR    ATAAGAAGAC TTTGACTCAG CATGCGGATG ACCTAAAAAC TTTGATCAGC TCCGCCATTA CCAACCAAGA 560
AryANE_v1_00021521   ---------- ---------- ---------- ---------- ---------- ---------- ---------- -

                                          580                   600                   620
MdPME2               AACTTGCCTT AATGGGTTCT CTCATGATGG AGGTGATAAG AAAGTTAGGA AAGTTTTGTT GGCTGGTGAG 630
MDP245813_cds_GDR    AACTTGCCTT AATGGGTTCT CTCATGATGG AGGTGATAAG AAAGTTAGGA AAGTTTTGTT GGCTGGTGAG 630
MDP222620_cds_GDR    AACTTGCCTT GATGGGTTCT CGCACGATGA TGCTGATAAA AAAGTTAGGA AAGTTTTTTT GGCCGGTCAG 630
AryANE_v1_00021521   ---------- ---------- ---------- ---------- ---------- ---------- ---------- -

                               640                   660                   680                   700
MdPME2               ---------- ---------- ---------- ---------- ---------- -GAACACATA GAAAAATTGT 649
MDP245813_cds_GDR    ACCGTCRATA TYTCTKTTAA CACCGATATT TRAACAATTG GCGTCACTCA GGAACACATA GAAAAATTGT 700
MDP222620_cds_GDR    ---------- ---------- ---------- ---------- ---------- -GTGCACGTA GAAAAATTGT 649
AryANE_v1_00021521   ---------- ---------- ---------- ---------- ---------- ---------- ---------- -

                                          720                   740                   760
MdPME2               GCAGTAATGT ACTGGCCATG ATCAAGAACA TGACCGATAC CGACATTGCA AACGAGATGA AAATGAATGT 719
MDP245813_cds_GDR    GCAGTAATGT ACTGGCCATG ATCAAGAACA TGACCGATAC CGACATTGCA AACGAGATGA AAATGAATGT 770
MDP222620_cds_GDR    GCAGTAATGC GCTGGCCATG ATCAAGAACA TGACCGATAC CGACATTGCA AACGAGATGA AAACGAATGT 719
AryANE_v1_00021521   ---------- ---------- ---------- ---------- ---------- ---------- ---------- -

                               780                   800                   820                   840
MdPME2               GAACCGAAAA CTCAAGGAGA ATATGGTGGA CGAGCACGGG TGGCCGGAGT GGCTGTCGGT GGCGGACAGG 789
MDP245813_cds_GDR    GAACCGAAAA CTCAAGGAGA ATATGGTGGA CGAGCACGGG TGGCCGGAGT GGCTGTCGGT GGCGGACAGG 840
MDP222620_cds_GDR    GAACCGAAAG CTGAAGGAGG ATGTGGTGGA CGAGCATGGG TGGCCGGAGT GGCTGTCGGT GGCAGACAGG 789
AryANE_v1_00021521   ---------- ---------- ---------- ---------- ---------- ---------- ---------- -

                                          860                   880                   900
MdPME2               CGACTGCTGC AGTCGTCTTC TGTCACGCCG AATGTGGTGG TGGCAGCAGA CGGCACTGGG AACTACAAGA 859
MDP245813_cds_GDR    CGACTGCTGC AGTCGTCTTC TGTCACGCCG AATGTGGTGG TGGCAGCAGA CGGCACTGGG AACTACAAGA 910
MDP222620_cds_GDR    CGACTGCTGC AGTCGTCATC TGTCACACCA AATGTGGTGG TGGCAGCAGA CGGGAGTGGG AACTACAAGA 859
AryANE_v1_00021521   ---------- ---------- ---------- ---------- ---------- ---------- ---------- -

                               920                   940                   960                   980
MdPME2               CGGTGTCGGA AGCGGTGGCT GCTGCCCCAG AGAAGAGTAG TAGCAGATAC GTGATTAAAA TAAAGGCGGG 929
MDP245813_cds_GDR    CGGTGTCGGA AGCGGTGGCT GCTGCCCCAG AGAAGAGTAG TAGCAGATAC GTGATTAAAA TAAAGGCGGG 980
MDP222620_cds_GDR    CGGTGTCGGC TGCGGTGGCT GCCGCCCCGG AGAAGAGCAG TAAAAGATAT GTGATTAGAA TAAAGGCGGG 929
AryANE_v1_00021521   ---------- ---------- ---------- ---------- ---------- ---------- ---------- -

                                        1 000                 1 020                 1 040
MdPME2               GATTTATAGA GAAAATGTGG ACGTGCCAAA GAAAAAGACC AACATTATGT TTTTGGGAGA TGGGAGGGTA 999
MDP245813_cds_GDR    GATTTATAGA GAAAATGTGG ACGTGCCAAA GAAAAAGACC AACATTATGT TTTTGGGAGA TGGGAGGGTA 1050
MDP222620_cds_GDR    GGTTTATAGA GAAAATGTGG ACGTGCCGAA GAAAAAGACC AACATTATGT TTATGGGAGA TGGGAGAAAA 999
AryANE_v1_00021521   ---------- ---------- ---------- ---------- ---------- ---------- ---------- -

                             1 060                 1 080                 1 100                 1 120
MdPME2               AACACCATCA TCACTGCTAG TAGAAATGTC GTCGATGGCA GCACAACTTT CAACTCAGCC ACAGTTGC-- 1067
MDP245813_cds_GDR    AACACCATCA TCACTGCTAG TAGAAATGTC GTCGATGGCA GCACAACTTT CAACTCAGCC ACAGTTGC-- 1118
MDP222620_cds_GDR    AACACTATCA TCACTGCTAG TAAAAATGTG GTCGATGGCA GCACAACTTT CAACTCGGCC ACAGTTGTGA 1069
AryANE_v1_00021521   ---------- ---------- --AAAATGTG GTCGATGGCA GCACAACTTT CAACTCGGCC ACAGTTGTGA 48
```

```
                                           1 140                   1 160                   1 180
            MdPME2 TGCG-GT-TG G----TGAGA AGTTTCTAGC CCGAGACATA ACATTCCAAA ACACAGCAGG CCCATCAAAG 1131
MDP245813_cds_GDR TGCG-GT-TG G----TGAGA AGTTTCTAGC CCGAGACATA ACATTCCAAA ACACAGCAGG CCCATCAAAG 1182
MDP222620_cds_GDR TGTATGTATG TACMTTGAGG ATTTTTT--- TCGGAWTCT- -C-TTTTGGA TCTTAG---- ---------- 1119
AryANE_v1_00021521 TGTATGTATG TA-------- ---------- ---------- ---------- ---------- ---------- 60
                          1 200                   1 220                   1 240                 1 260
            MdPME2 CACCAAGCTG TTGCCCTAAG AGTTGGGTCC GATCTCTCAG CATTCTACCG CTGCGACATT CTTGCGTACC 1201
MDP245813_cds_GDR CACCAAGCTG TTGCCCTAAG AGTTGGGTCC GATCTCTCAG CATTCTACCG CTGCGACATT CTTGCGTACC 1252
MDP222620_cds_GDR ---------- ---------- ---------- ---------- ---------- ---------- ---------- 1119
AryANE_v1_00021521 ---------- ---------- ---------- ---------- ---------- ---------- ---------- 60
                                           1 280                   1 300                   1 320
            MdPME2 AAGACTCGCT CTACGTGCAC TCGAATCGCC AGTTTTTTGA AGGCTGCTTG GTAGCCGGAA CAGTTGACTT 1271
MDP245813_cds_GDR AAGACTCGCT CTACGTGCAC TCGAATCGCC AGTTTTTTGA AGGCTGCTTG GTAGCCGGAA CAGTTGACTT 1322
MDP222620_cds_GDR ---------- ---------- ---------- ---------- ---------- ---------- ---------- 1119
AryANE_v1_00021521 ---------- ---------- ---------- ---------- ---------- ---------- ---------- 60
                          1 340                   1 360                   1 380                 1 400
            MdPME2 CATCTTCGGC AATGCTGCTG TCGTGTTACA AAATTGTGAC ATCCATGCAA GAAAACCTGA TTCCGGCCAG 1341
MDP245813_cds_GDR CATCTTCGGC AATGCTGCTG TCGTGTTACA AAATTGTGAC ATCCATGCAA GAAAACCTGA TTCCGGCCAG 1392
MDP222620_cds_GDR ---------- ---------- ---------- ---------- ---------- ---------- ---------- 1119
AryANE_v1_00021521 ---------- ---------- ---------- ---------- ---------- ---------- ---------- 60
                                           1 420                   1 440                   1 460
            MdPME2 AAGAACATGG TCACTGCACA AGGTAGAACT GACCCGAATC AAAACACCGG CATCGTGATC CAAAAATCGA 1411
MDP245813_cds_GDR AAGAACATGG TCACTGCACA AGGTAGAACT GACCCGAATC AAAACACCGG CATCGTGATC CAAAAATCGA 1462
MDP222620_cds_GDR ---------- ---------- ---------- ---------- ---------- ---------- ---------- 1119
AryANE_v1_00021521 ---------- ---------- ---------- ---------- ---------- ---------- ---------- 60
                          1 480                   1 500                   1 520                 1 540
            MdPME2 GAATCGGCGC CACTTCCGAC TTGCAAGCCT CAAAGAGCAG CTTCAAAACG TTTTTGGGGA GGCCATGGAA 1481
MDP245813_cds_GDR GAATCGGCGC CACTTCCGAC TTGCAAGCCT CAAAGAGCAG CTTCAAAACG TTTTTGGGGA GGCCATGGAA 1532
MDP222620_cds_GDR ---------- ---------- ---------- ---------- ---------- ---------- ---------- 1119
AryANE_v1_00021521 ---------- ---------- ---------- ---------- ---------- ---------- ---------- 60
                                           1 560                   1 580                   1 600
            MdPME2 GGAGTATTCA AGGACTGTGA TCATGCAGTC GAGTATAACT GATATCATTG ACCCTGCTGG ATGGCATGAG 1551
MDP245813_cds_GDR GGAGTATTCA AGGACTGTGA TCATGCAGTC GAGTATAACT GATATCATTG ACCCTGCTGG ATGGCATGAG 1602
MDP222620_cds_GDR ---------- ---------- ---------- ---------- ---------- ---------- ---------- 1119
AryANE_v1_00021521 ---------- ---------- ---------- ---------- ---------- ---------- ---------- 60
                          1 620                   1 640                   1 660                 1 680
            MdPME2 TGGTCTGGCA CTTTTGCCCT CGACACTTTG TTTTATGGAG AGTATGCTAA CACTGGGGCA GGCGCTGGGA 1621
MDP245813_cds_GDR TGGTCTGGCA CTTTTGCCCT CGACACTTTG TTTTATGGAG AGTATGCTAA CACTGGGGCA GGCGCTGGGA 1672
MDP222620_cds_GDR ---------- ---------- ---------- ---------- ---------- ---------- ---------- 1119
AryANE_v1_00021521 ---------- ---------- ---------- ---------- ---------- ---------- ---------- 60
                                           1 700                   1 720                   1 740
            MdPME2 CTGCAAACAG GGTGACGTGG AAGGGGTTTA AGGTAATTAC AAGTCCCACT GAGGCTGAGG CGTATACGCC 1691
MDP245813_cds_GDR CTGCAAACAG GGTGACGTGG AAGGGGTTTA AGGTAATTAC AAGTCCCACT GAGGCTGAGG CGTATACGCC 1742
MDP222620_cds_GDR ---------- ---------- ---------- ---------- ---------- ---------- ---------- 1119
AryANE_v1_00021521 ---------- ---------- ---------- ---------- ---------- ---------- ---------- 60
                          1 760                   1 780                   1 800
            MdPME2 GGGCAACTTT ATTGCTGGTG GTAGTTGGTT GAGTTCTACC GGTTTTCCAT TTTCTCTTGC GCTGTAA 1758
MDP245813_cds_GDR GGGCAACTTT ATTGCTGGTG GTAGTTGGTT GAGTTCTACC GGTTTTCCAT TTTCTCTTGC GCTGTAA 1809
MDP222620_cds_GDR ---------- ---------- ---------- ---------- ---------- ---------- ------- 1119
AryANE_v1_00021521 ---------- ---------- ---------- ---------- ---------- ---------- ------- 60
```
