# Supplementary material for: Multiscale investigation of mealiness in apple: an atypical role for a pectin methylesterase during fruit maturation
Source: BMC Plant Biol. 2014 Dec 31;14:375. doi: 10.1186/s12870-014-0375-3 (PMC4310206; doi:10.1186/s12870-014-0375-3)

Additional file 6. Evolutionary relationships of 60 apple PMEs.

The tree is based on the alignment of protein sequences using MUSCLE and it is constructed using the neighbor-joining method and a bootstrap test with 1000 iterations using MEGA5 software. Black lines grouped PME genes of type I, including *MdPME2*, and gray are of type II according to *Arabidopsis thaliana* PME groups (Pelloux *et al*., 2007).

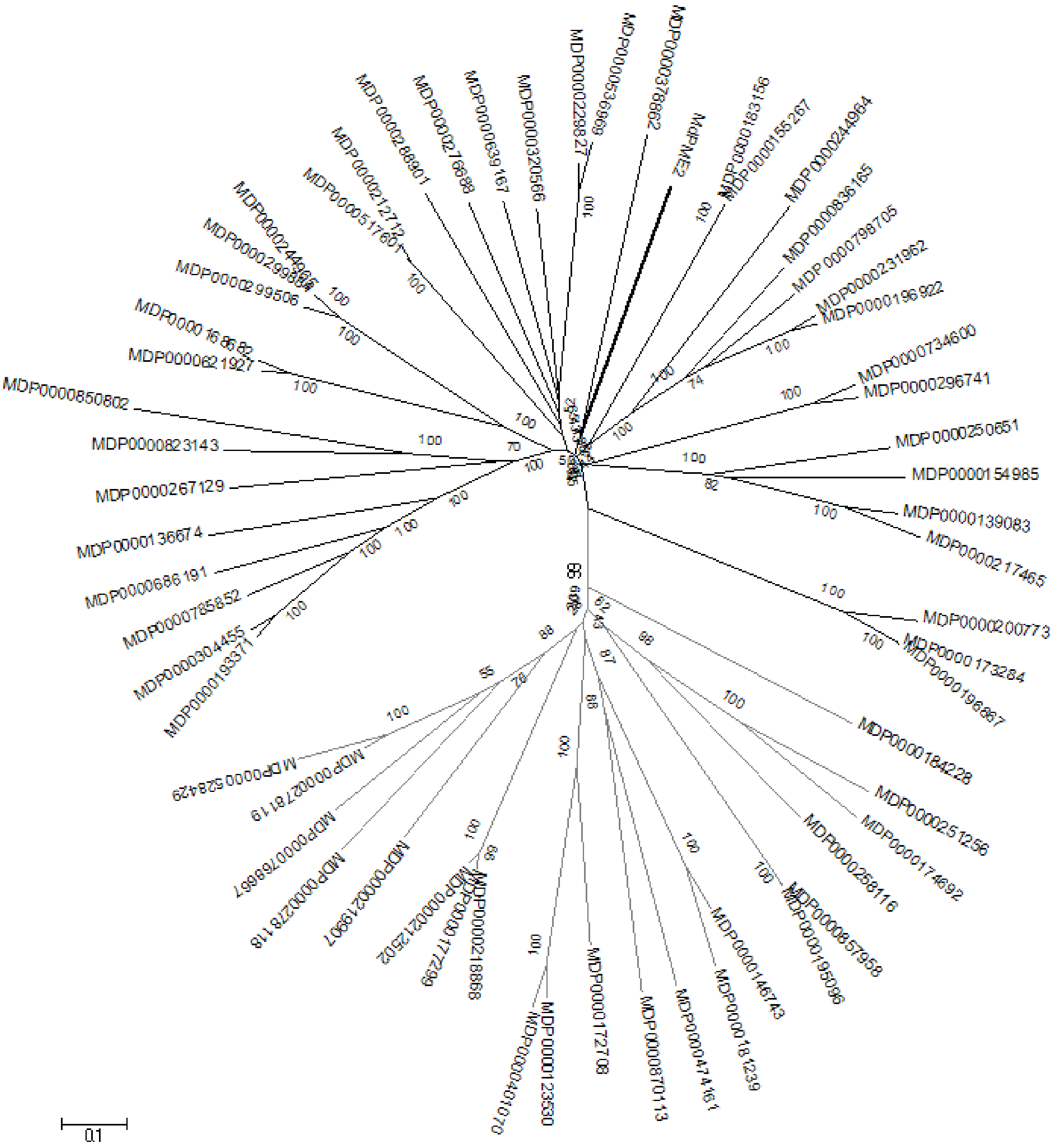

Supplement: Additional file 6: — Evolutionary relationships between 60 apple PMEs. The tree is based on the alignment of protein sequences using MUSCLE and it is constructed using the neighbor-joining method and a bootstrap test with 1000 iterations using MEGA5 software. Black lines grouped PME genes of type I, including MdPME2, and gray are of type II according to Arabidopsis thaliana PME groups [33]. [file 12870_2014_375_MOESM6_ESM.pdf]
