# Supplementary material for: Multiscale investigation of mealiness in apple: an atypical role for a pectin methylesterase during fruit maturation
Source: BMC Plant Biol. 2014 Dec 31;14:375. doi: 10.1186/s12870-014-0375-3 (PMC4310206; doi:10.1186/s12870-014-0375-3)

Additional file 7. Degree of methylesterification (DM) of apple cell walls (2009, 2010) for mealy (M40, M74) and non-mealy hybrids (M20, M49) during the kinetic.

Values are the means of 3 technical replicates for both years. Error bars represent the standard deviation.

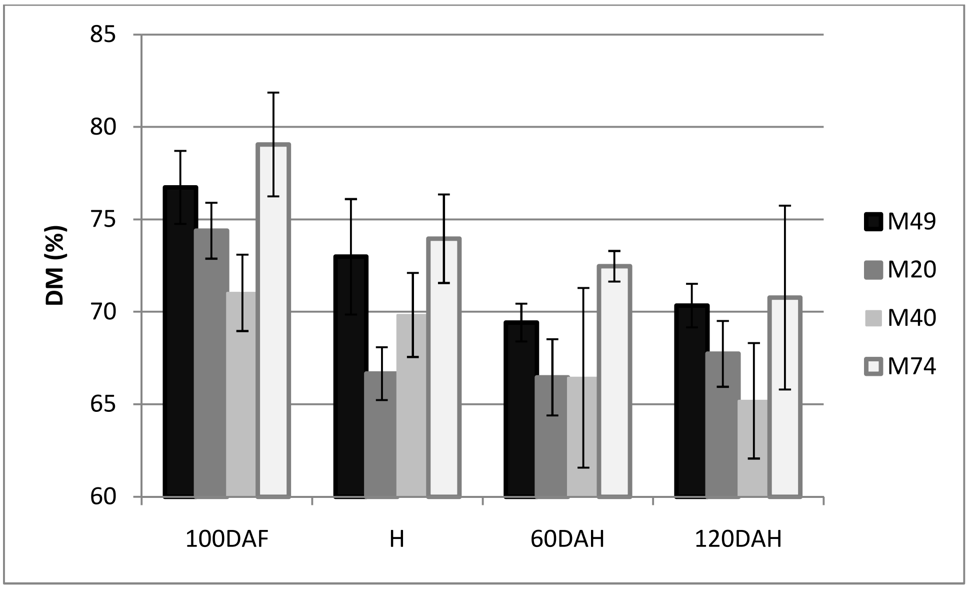

Supplement: Additional file 7: — Degree of methylesterification (DM) of apple cell walls (2009, 2010) for mealy (M40, M74) and non-mealy hybrids (M20, M49) during the kinetic. Values are the means of 3 technical replicates for both years. Error bars represent the standard deviation. [file 12870_2014_375_MOESM7_ESM.pdf]
