# Supplementary material for: Multiscale investigation of mealiness in apple: an atypical role for a pectin methylesterase during fruit maturation
Source: BMC Plant Biol. 2014 Dec 31;14:375. doi: 10.1186/s12870-014-0375-3 (PMC4310206; doi:10.1186/s12870-014-0375-3)

## Additional file 8. Pectin enzymatic profiling results.

**S8.A Sum up of significant differences between mealy and non-mealy fruits for each oligouronide during the complete apple fruit cycle according to Kruskall-Wallis tests.**

The oligouronides were identified from MALDI-TOF MS spectra after pectin-lyase hydrolysis of cell wall preparations. Oligouronides nomenclature is described in the materials and methods. Colors refer to significant differences (p-value<0.05) between mealy and non-mealy hybrids: dark grey is for a higher abundance in mealy fruits, light grey: a higher abundance in non-mealy fruits.

**S8.B Bar charts of abundance of each oligouronides for mealy (M40, M74) and non-mealy (M49, M74) hybrids at the four fruit developmental stages** obtained from MALDI-TOF MS spectra after pectin-lyase hydrolysis of cell wall preparations. Oligouronides nomenclature is described in the materials and methods. Letters on bar charts indicate groups of significantly different abundance (Kruskall-Wallis test). M: mealy; NM: non-mealy.

**S8.A**

| oligourinodes | 100 DAF | H | 60 DAH | 120 DAH |
|---|---|---|---|---|
| DU4m2 | | | | |
| DU4m3 | | | | |
| DU5m3 | | | | |
| DU5m4 | | | | |
| DU4m3a1 | | | | |
| DU6m1a1 | | | | |
| DU6m2a1 | | | | |
| DU7m2a1 | | | | |
| DU7m3a1 | | | | |
| DU6m3a1 | | | | |
| DU5m2a1 | | | | |
| DU5m4a1 | | | | |
| DU6m2a2 | | | | |
| DU6m4a1 | | | | |
| DU7m3a2 | | | | |

**S8.B**

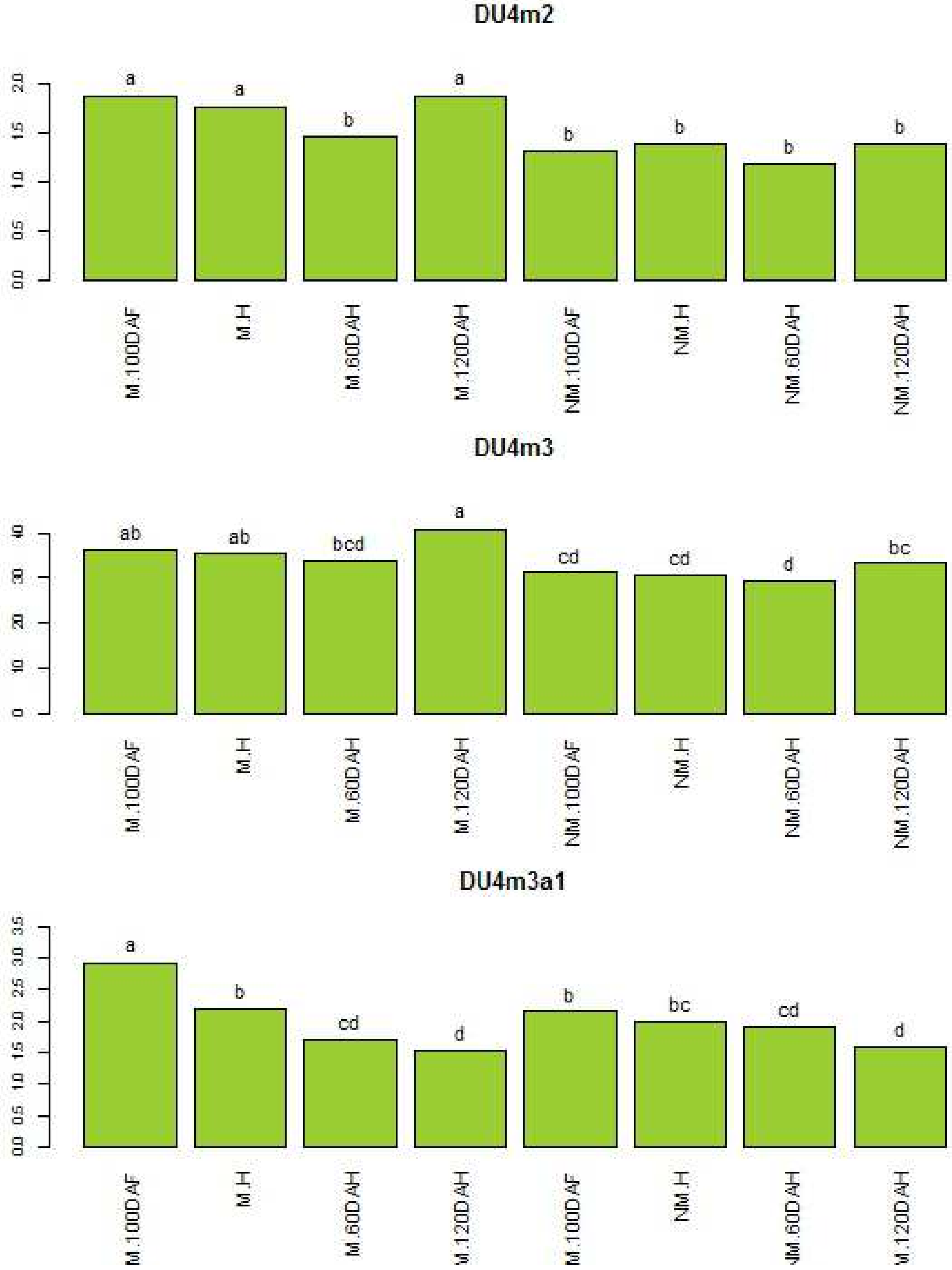
DU4m2
0.0
0.5
1.0
1.5
2.0
a
a
b
a
b
b
b
b
M.100DAF
M.H
M.60DAH
M.120DAH
NM.100DAF
NM.H
NM.60DAH
NM.120DAH
DU4m3
0
10
20
30
40
ab
ab
bcd
a
cd
cd
d
bc
M.100DAF
M.H
M.60DAH
M.120DAH
NM.100DAF
NM.H
NM.60DAH
NM.120DAH
DU4m3a1
0.0
0.5
1.0
1.5
2.0
2.5
3.0
3.5
a
b
cd
d
b
bc
cd
d
M.100DAF
M.H
M.60DAH
NM.H

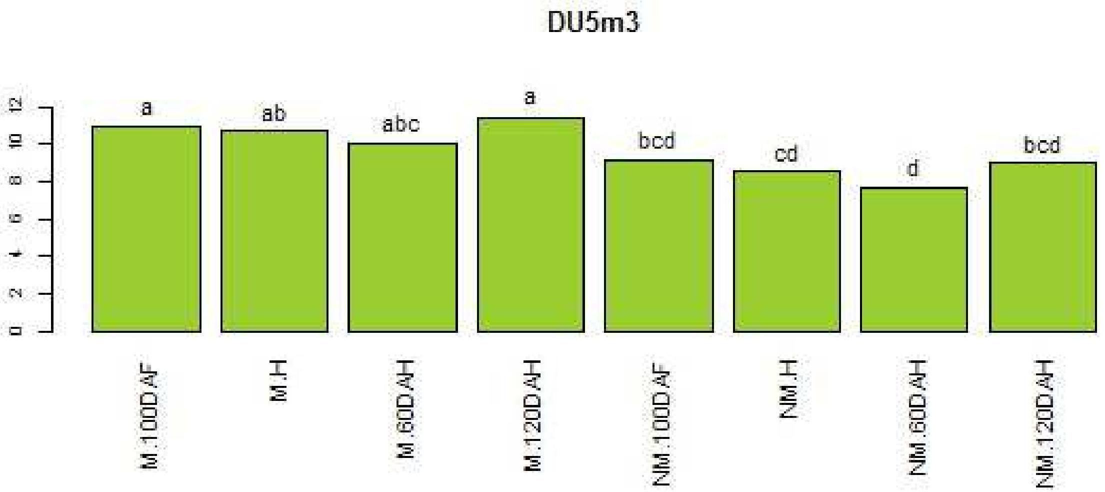
DU5m3
0
2
4
6
8
10
12
a
ab
abc
a
bcd
cd
d
bcd
M.100DAF
M.H
M.60DAH
M.120DAH
NM.100DAF
NM.H
NM.60DAH
NM.120DAH

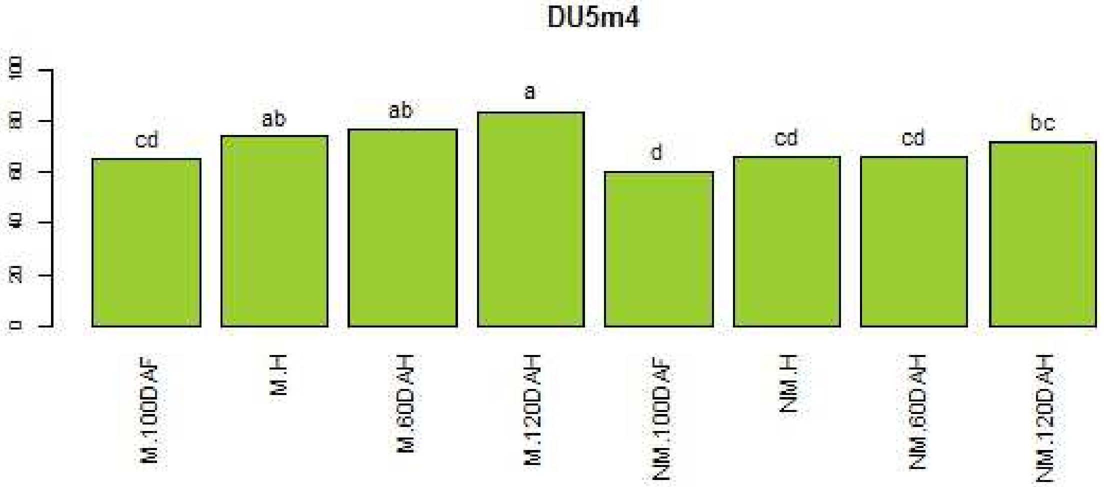
DU5m4
0
20
40
60
80
100
cd
ab
ab
a
d
cd
cd
bc
M.100DAF
M.H
M.60DAH
M.120DAH
NM.100DAF
NM.H
NM.60DAH
NM.120DAH

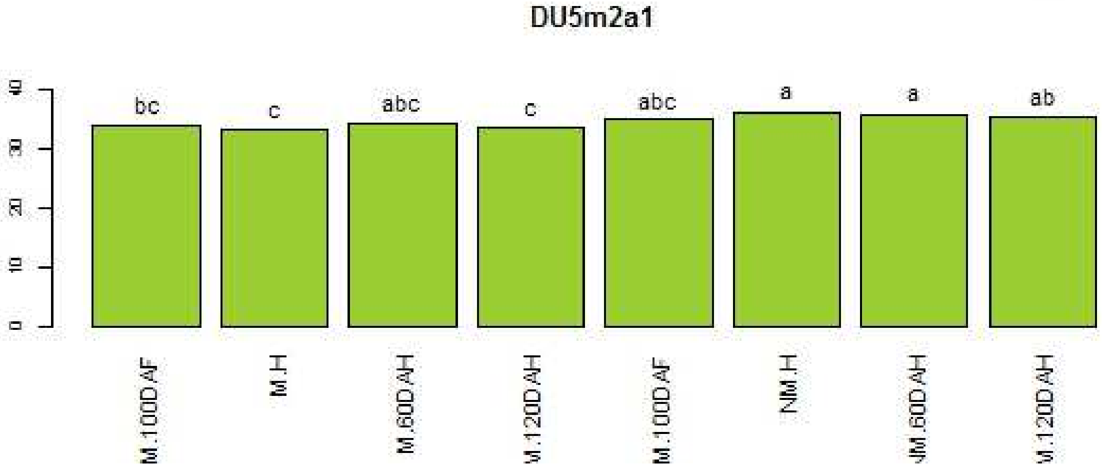
DU5m2a1
0
10
20
30
40
bc
c
abc
c
abc
a
a
ab
M.100DAF
M.H
M.60DAH
M.100DAF
NM.H

## DU5m4a1

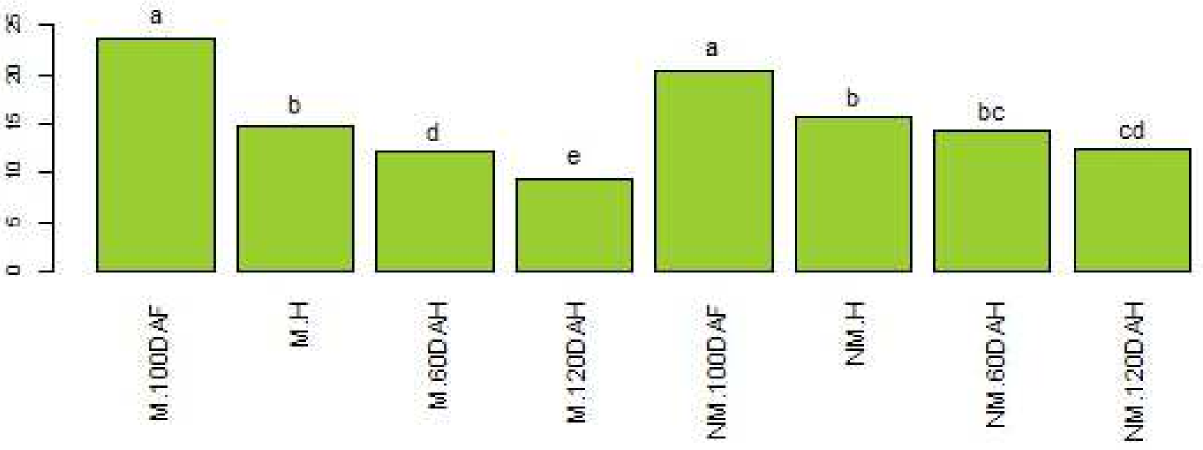


## DU6m1a1

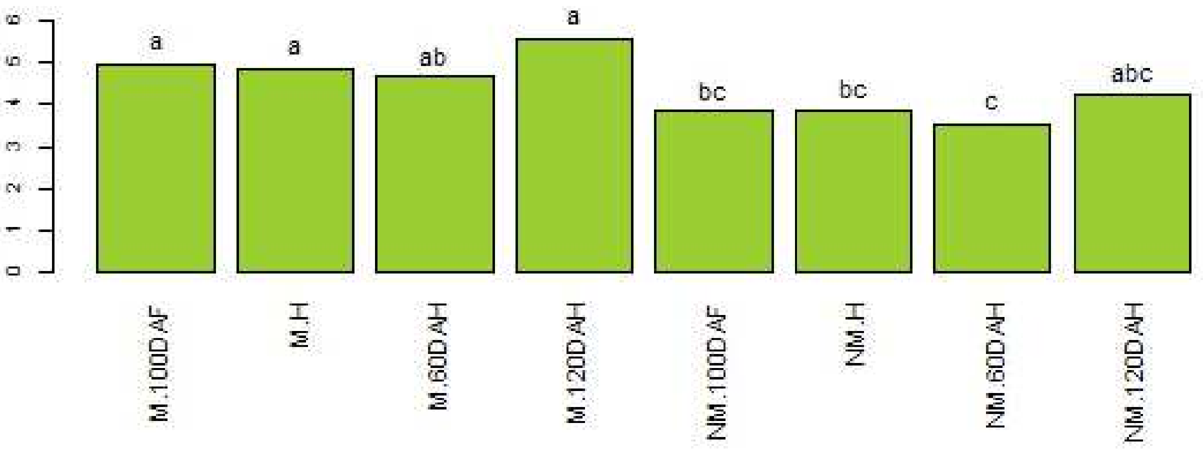


## DU6m2a1

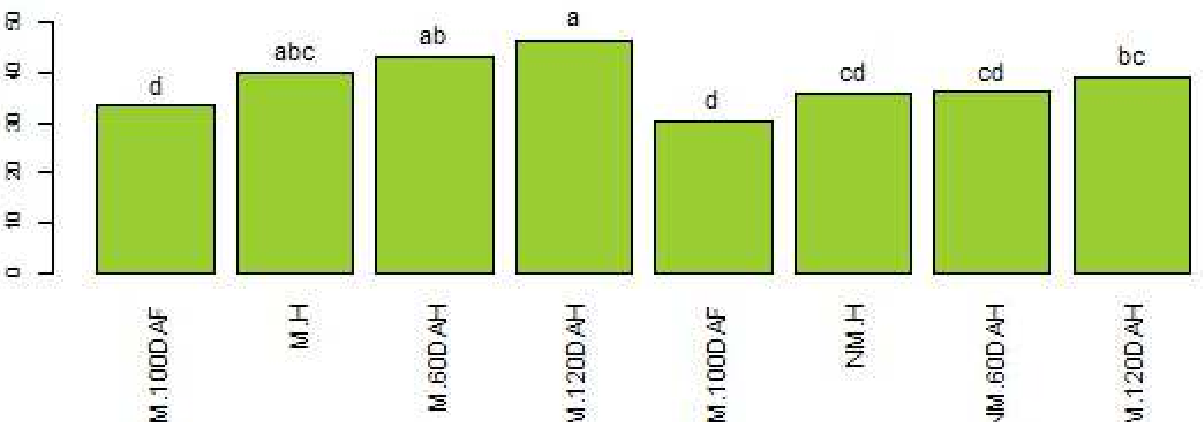

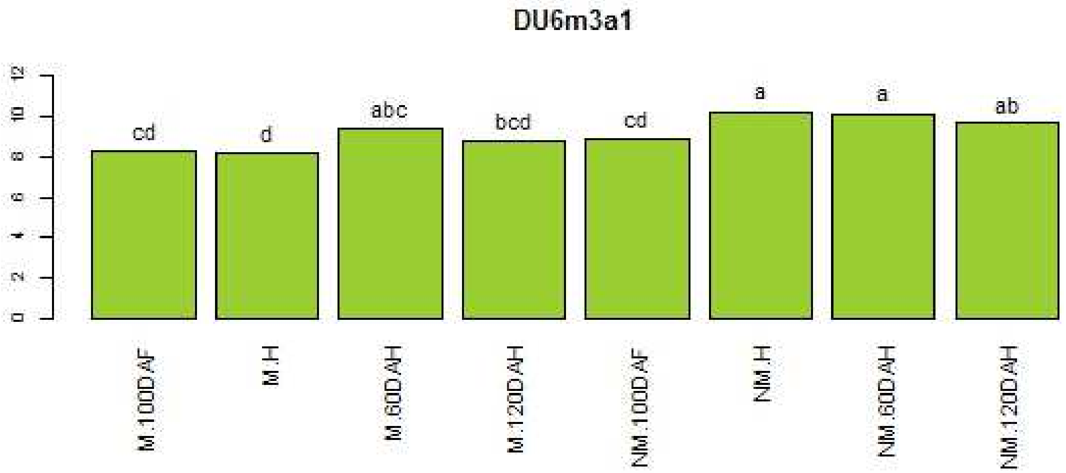
DU6m3a1
0
2
4
6
8
10
12
cd
d
abc
bcd
cd
a
a
ab
M.100DAF
M.H
M.60DAH
M.120DAH
NM.100DAF
NM.H
NM.60DAH
NM.120DAH

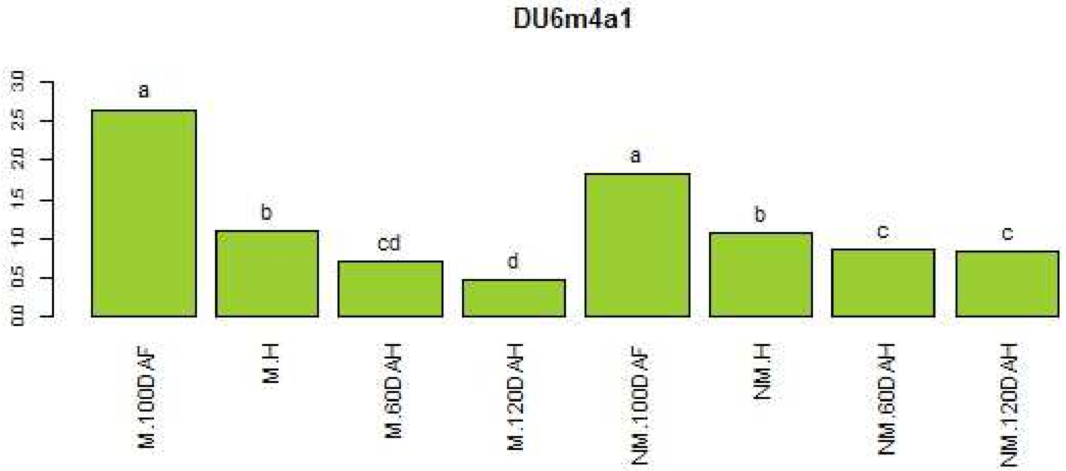
DU6m4a1
0.0
0.5
1.0
1.5
2.0
2.5
3.0
a
b
cd
d
a
b
c
c
M.100DAF
M.H
M.60DAH
M.120DAH
NM.100DAF
NM.H
NM.60DAH
NM.120DAH

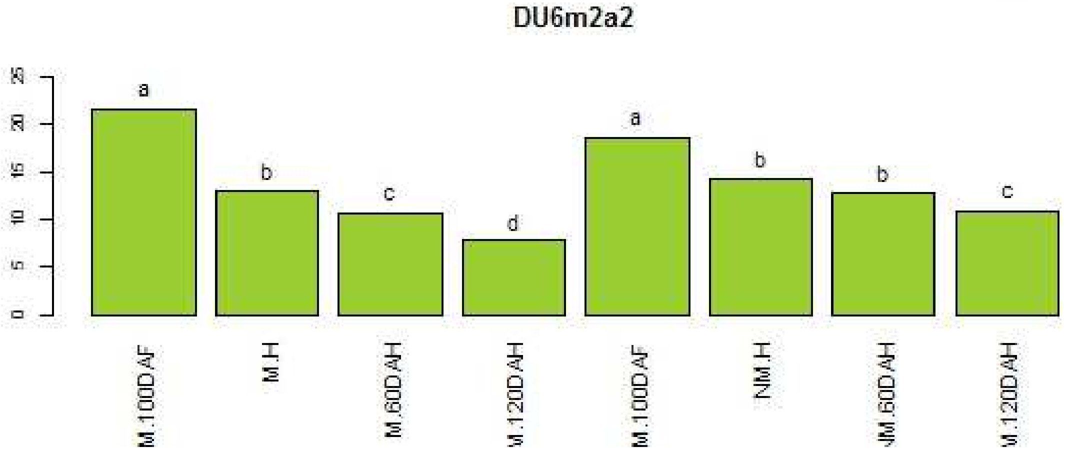
DU6m2a2
0
5
10
15
20
25
a
b
c
d
a
b
b
c
M.100DAF
M.H
M.60DAH
M.120DAH
M.100DAF
NM.H
NM.60DAH
M.120DAH

**DU7m2a1**

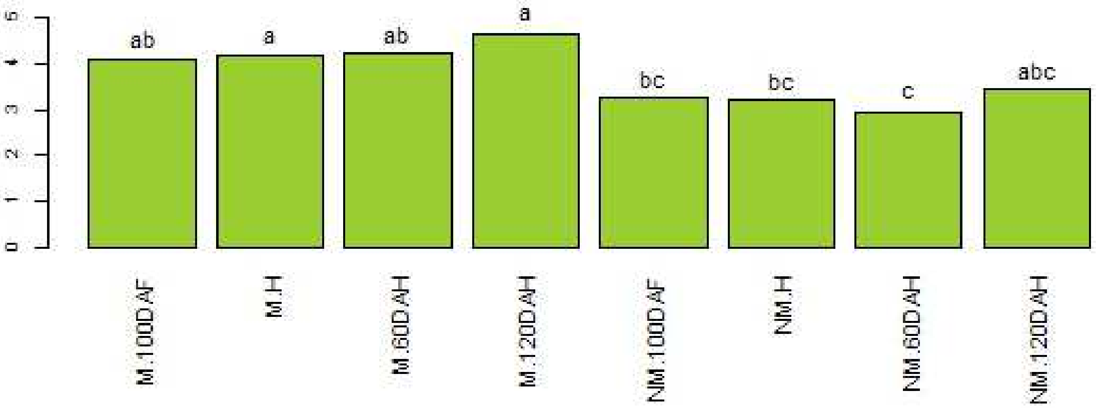


**DU7m3a1**

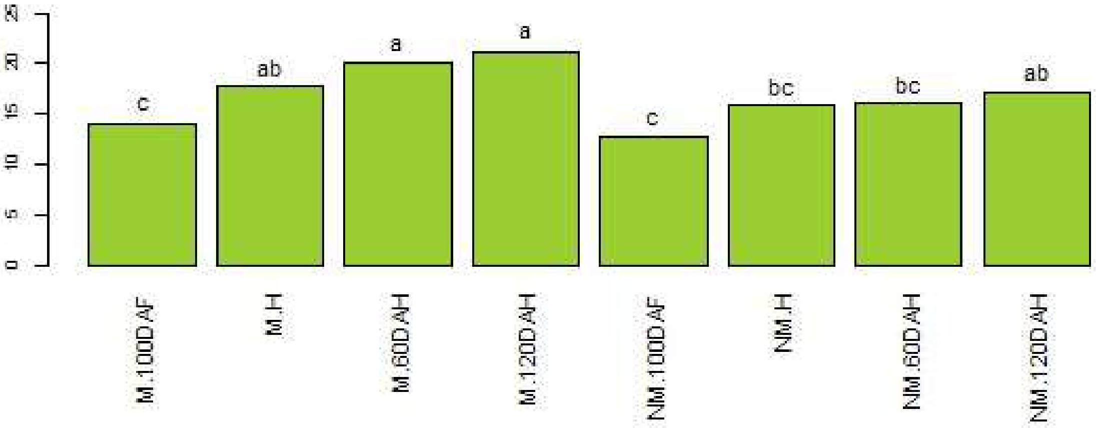


**DU7m3a2**

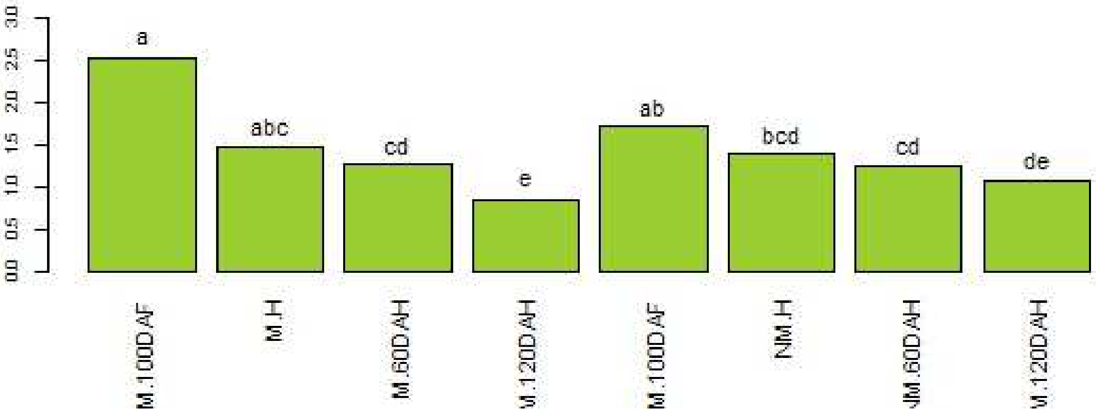

Supplement: Additional file 8: — Pectin enzymatic profiling results. S8.A Sum up of significant differences between mealy and non-mealy fruits for each oligouronide during the complete apple fruit cycle according to Kruskall-Wallis tests. The oligouronides were identified from MALDI-TOF MS spectra after pectin-lyase hydrolysis of cell wall preparations. Oligouronides nomenclature is described in the materials and methods. Colors refer to significant differences (p-value < 0.05) between mealy and non-mealy hybrids: dark grey is for a higher abundance in mealy fruits, light grey: a higher abundance in non-mealy fruits. S8.B Bar charts of abundance of each oligouronides for mealy (M40, M74) and non-mealy (M49, M74) hybrids at the four fruit developmental stages obtained from MALDI-TOF MS spectra after pectin-lyase hydrolysis of cell wall preparations. Oligouronides nomenclature is described in the materials and methods. Letters on bar charts indicate groups of significantly different abundance (Kruskall-Wallis test). M: mealy; NM: non-mealy. [file 12870_2014_375_MOESM8_ESM.pdf]
