# Supplementary figures and images for: Multiscale investigation of mealiness in apple: an atypical role for a pectin methylesterase during fruit maturation
Source: BMC Plant Biol. 2014 Dec 31;14:375. doi: 10.1186/s12870-014-0375-3 (PMC4310206; doi:10.1186/s12870-014-0375-3)

Additional file 9. IM population within HIDRAS pedigree (adapted from Kouassi *et al.*, 2009).

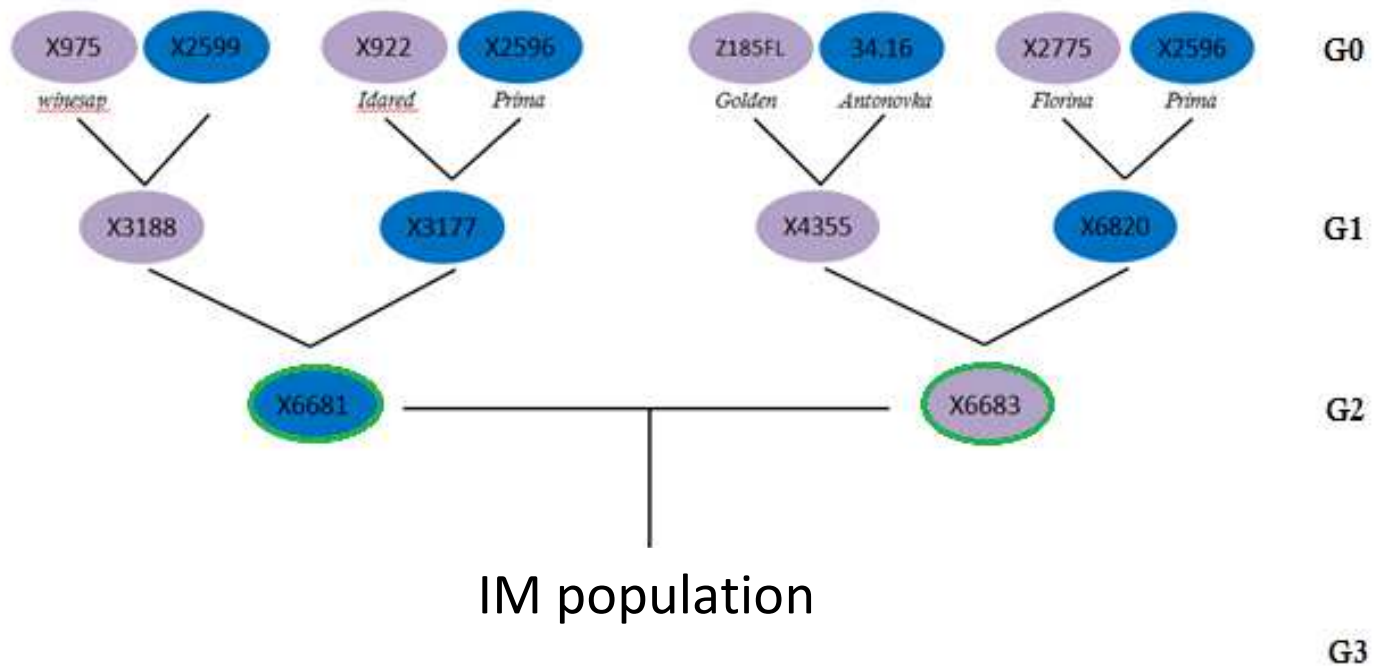

Supplement: Additional file 9: — IM population within HIDRAS pedigree [ 24 ]. [file 12870_2014_375_MOESM9_ESM.pdf]
