# Supplementary material for: Multiscale investigation of mealiness in apple: an atypical role for a pectin methylesterase during fruit maturation
Source: BMC Plant Biol. 2014 Dec 31;14:375. doi: 10.1186/s12870-014-0375-3 (PMC4310206; doi:10.1186/s12870-014-0375-3)
Supplement: Additional file 10: — List of primers used for RT-qPCR and cloning. S10.A Primers used for the RT-qPCR. For each selected gene, the expression pattern obtained by microarray analysis is specified (up or down in mealy fruits compared to non-mealy). Three housekeeping genes (HK) were used to calculate the normalisation factor. S10.B Primers used for MdPME2 cloning. [file 12870_2014_375_MOESM10_ESM.pdf]

Additional file 10. List of primers used for RT-qPCR and cloning.

S10.A Primers used for the RT-qPCR. For each selected gene, the expression pattern obtained by microarray analysis is specified (up or down in mealy fruits compared to non-mealy). Three housekeeping genes (HK) were used to calculate the normalisation factor.

S10.B Primers used for *MdPME2* cloning.

S10.A

Application	Expression profil	MDP number	Gene annotation	Size	Sequence (5' → 3')
RT PCR	Down	MDP0000222620	AtPME2	104	F: TTGAAAGTTGTGCTGCCATC<br>R: GACGTGCCGAAGAAAAAGAC
RT PCR	Down	MDP0000322658	UDP-glucoronosyl	100	F: TTGAATCAGCTTGTCGAGTTGT<br>R: GGAATCATAAGCTTCCCAGATTT
RT PCR	Down	MDP0000393227	FQR1	184	F: GCATAAGTTCCCGCACCATA<br>R: CCGCTGGAATTTTCTACAGC
RT PCR	Down	MDP0000671440	GDH1	102	F: GCAAACGCTTGAGATCTTCG<br>R: AGCACAGCTGATGACATGGA
RT PCR	Up	MDP0000303194	CRK10	150	F: TGTTGATCTTCCAGTACCCTCA<br>R: CCATGCAGCAGATATTTGACA
RT PCR	HK	MDP0000271281	porin, putative	155	F: CGCCCAAAATTATTGAAGCA<br>R: ATCCCCAGCCATTGATTTTT
RT PCR	HK	MDP0000217860	drought-responsive family protein	193	F: ACGCAAGAGGAAGACAAGGA<br>R: GGGTGGCATGCTAACAAAAT
RT PCR / sqPCR	HK	MDP0000645828	GADPH	165	F: ATCAATGAAGGACTGGAGAGG<br>R: GTCAGGTCAACAACGGAAAC

S10.B

Application	MDP number	Gene annotation	Sequence (5' → 3')
sq PCR	MDP0000222620	AtPME2	F: TCACGATGCTGTGGAGGAC<br>R: TGAAAGTTGTGCTGCCATCG
sq PCR	MDP0000245813		F: CCACGAGGCTGTCGAGGAT<br>R: TGAAAGTTGTGCTGCCATCG
3' RACE	MdPME2	PME	F6: CGATGGCAGCACAACTTTCA
PCR1	MdPME2	PME	F5: AGAATGGCCAAAATCAAAGAGT
nested PCR	MdPME2	PME	F1: CACCAGAATGGCCAAAATCAAAGAG
	MdPME2	PME	R: CAGCGCAAGAGAAAATGGAA
